# Systematic analysis of mistletoe prescriptions in clinical studies

**DOI:** 10.1007/s00432-022-04511-2

**Published:** 2022-12-09

**Authors:** Henrike Staupe, Judith Buentzel, Christian Keinki, Jens Buentzel, Jutta Huebner

**Affiliations:** 1 Klinik Für Innere Medizin II, Hämatologie Und Onkologie, Universitätsklinikum Jena, Jena, Germany; 2 Klinik Für Hämatologie Und Medizinische Onkologie, Universitätsmedizin Göttingen, Göttingen, Germany; 3 Klinik Für HNO-Erkrankungen, Südharz-Klinikum Nordhausen, Nordhausen, Germany

**Keywords:** Mistletoe, Cancer, Complementary and alternative medicine (CAM)

## Abstract

**Purpose:**

Mistletoe treatment is discussed controversial as a complementary treatment for cancer patients. Aim of this systematic analysis is to assess the concept of mistletoe treatment in the clinical studies with respect to indication, type of mistletoe preparation, treatment schedule, aim of treatment, and assessment of treatment results.

**Methods:**

In the period from August to December 2020, the following databases were systematically searched: Medline, Embase, Cochrane Central Register of Controlled Trials (CENTRAL), PsycINFO, CINAHL, and “Science Citation Index Expanded” (Web of Science). We assessed all studies for study types, methods, endpoints and mistletoe preparations including their ways of application, host trees and dosage schedules.

**Results:**

The search concerning mistletoe therapy revealed 3296 hits. Of these, 102 publications and at total of 19.441 patients were included. We included several study types investigating the application of mistletoe in different groups of participants (cancer patients of any type of cancer were included as well as studies conducted with healthy volunteers and pediatric patients). The most common types of cancer were breast cancer, pancreatic cancer, colorectal cancer and malignant melanoma. Randomized controlled studies, cohort studies and case reports make up most of the included studies. A huge variety was observed concerning type and composition of mistletoe extracts (differing pharmaceutical companies and host trees), ways of applications and dosage schedules. Administration varied e. g. between using mistletoe extract as sole treatment and as concomitant therapy to cancer treatment. As the analysis of all studies shows, there is no relationship between mistletoe preparation used, host tree and dosage, and cancer type.

**Conclusions:**

Our research was not able to deviate transparent rules or guidelines with respect to mistletoe treatment in cancer care.

**Supplementary Information:**

The online version contains supplementary material available at 10.1007/s00432-022-04511-2.

## Background

Mistletoe extracts are used in cancer patients either as sole alternative therapy or commonly as complementary treatment in addition to conventional cancer therapy (Ebel et al. 2015; Huebner et al. 2014).

Mistletoe (Viscum album) is a small plant growing as a hemiparasite on several types of host trees in Europe, Asia and North Africa (Becker 1986). Mistletoe host trees include, for example fir (Abietis), maple (Aceris), almond (Amygdali), birch (Betulae), hawthorn (Crategi), ash (Fraxini), apple tree (Mali), pine (Pini), poplars (Populi) and oak (Quercus).

Two different groups of mistletoe preparations exist. Phytotherapeutic mistletoe preparations which are applied at a constant dose of lektines (Cefalektin^®^, Eurixor^®^ and Lektinol^®^) and anthroposophical or homeopathically produced mistletoe preparations (Helixor^®^, Iscador^®^, Abnobaviscum^®^, Iscucin^®^, Isorel^®^ and Plenosol^®^) are used in cancer treatment (Lange-Lindberg et al. 2006). In the second group, the dose of mistletoe preparation should be increased continuously depending on the general condition of the patient, the extent of the local reaction at the injection site and the regulation of body temperature (Horneber et al. 2008). According to the recommendations of the manufacturers, the preparations should usually be administered two to three times a week by subcutaneous injection in increasing dosages (Horneber et al. 2008). Mistletoe extracts are also increasingly administered intravenously, intratumorally, or intracavitarily (Kienle and Kiene 2007). These ways of application are off-label injections.

The various viscum extracts differ in their composition of the individual components (viscotoxins, lectins, polysaccharides, flavonoids, triterpenes and polypeptides), as well as the time of harvesting and different production processes (Holandino et al. 2020; Horneber et al. 2008; Nazaruk and Orlikowski 2016).

In cancer therapy, there are two discussed theses on mistletoe’s mode of clinical relevant action: First, some authors postulate that mistletoe extracts have a cytostatic, i.e., growth-inhibiting, and immunomodulating effect, i.e., immune system-stimulating effect via cytotoxic substances or via lectins (Boneberg and Hartung 2001; Felenda et al. 2019; Gardin 2009; Hajtó et al. 2005; Hostanska et al. 1995; Huber et al. 2006; Hulsen et al. 1989; Jurin et al. 1993; Klingbeil et al. 2013; Menke et al. 2019, 2020; Seifert et al. 2008; Tabiasco et al. 2002; Zhao et al. 2019). Second, some authors say that mistletoe improves well-being and quality of life and reduces side effects related to conventional cancer treatment (Beuth et al. 2008; Bussing et al. 2012; Cazacu et al. 2003; Eisenbraun et al. 2011; Horneber et al. 2008; Kienle and Kiene 2010; Kim et al. 2012; Lange-Lindberg et al. 2006; Loef and Walach 2020; Loewe-Mesch et al. 2008; Pelzer et al. 2022; Piao et al. 2004; Semiglazov et al. 2006), possibly triggered by the release of endorphins (Heiny and Beuth 1994; Lenartz et al. 1999).

The benefit of mistletoe treatment is still discussed and highly controversial. There are systematic reviews supporting the thesis that mistletoe extracts can improve quality of life in general (Bussing et al. 2012; Horneber et al. 2008; Loef and Walach 2020; Melzer et al. 2009), in breast cancer patients (Kienle et al. 2009; Kienle and Kiene 2010; Lange-Lindberg et al. 2006) and in patients with gynaecological cancer (Kienle et al. 2009). In the study of Pelzer et al. (2022) only a moderate effect on cancer-related fatigue of similar size as physical activity was reported. Other authors attribute a positive benefit to mistletoe therapy in terms of survival (Ostermann et al. 2009, 2020) and toxicity of the main intervention (Kienle et al. 2009). However, some of these reviews have a high risk of bias and heterogeneity (Loef and Walach 2020; Ostermann et al. 2020). In contrast, Horneber et al. (2008) and Lange-Lindberg et al. (2006) concluded that the available evidence from the included studies was insufficient to prove efficacy of mistletoe treatment regarding survival and reduction of the toxicity of chemotherapeutic treatment. In addition, Freuding et al. (2019a, b) also concluded that in terms of survival, quality of life or reduction of treatment-related side effects, there is no indication to prescribe mistletoe to patients with cancer. In the statement of Matthes et al. (2020), a retraction of the two-part review of Freuding et al. (2019a, b) is demanded due to a lack of methodological quality. According to the current German S3-Guideline on complementary medicine in the treatment of oncological patients, the administration of mistletoe preparations is controversial due to heterogeneous systematic reviews (Leitlinienprogramm Onkologie:Deutsche Krebsgesellschaft, Deutsche Krebshilfe, AWMF 2021).

In all these discussions, mistletoe is regarded as a concise treatment concept. So far, literature did not consider the different mistletoe preparations and treatment schedules. However, comparability between type of drug, applications and treatment schedules should be regarded as base for systematic reviews as well as meta-analyses while deliberating the role of mistletoe treatment in modern oncology.

### Objectives

Aim of this systematic analysis is assess the concept of mistletoe treatment in the clinical studies with respect to indication, type of mistletoe preparation, treatment schedule, aim of treatment, and assessment of treatment results.

## Methods

### Criteria for including studies in this review

Inclusion and exclusion criteria are listed in supplementary file 2 (Table e1). We included cancer patients as well as studies with healthy persons which assessed endpoints regarded as relevant for mistletoe treatment in cancer care (for example immunological parameters).

### Search strategy

In the period from August to December 2020, the following databases were systematically searched: Medline, Embase, Cochrane Central Register of Controlled Trials (CENTRAL), PsycINFO, CINAHL, and “Science Citation Index Expanded” (Web of Science). A complex search string was developed for each database. This contained a combination of mesh terms/keywords and text words related to cancer and mistletoe. To make sure that no study would be missed, the search was not limited by filters for study type. Only articles published in English or German were considered. The exact search strategy with the respective applied mesh terms/keywords and text words for each database is shown in supplementary file 3 (Table e2, Freuding et al. 2019a, b).

### Data collection and analysis

#### Selection of studies

##### Study selection of this review was made in several steps

First, titles and abstracts of all clinical and preclinical studies with descriptions of the respective study population, mistletoe preparation (name and dosage), endpoints and results were collected in a table and screened for relevance to this review by two independent reviewers (HS, JH). Reasons for rejecting studies were irrelevant topics, studies with other anthroposophic study medications than mistletoe preparations and studies no endpoint associated with cancer of cancer treatment.

Second, all articles were excluded that were not scientific articles published as full papers in a peer-reviewed journal.

After that, full texts from all remaining studies were screened and again it was decided by two independent reviewers (HS, JH) if they matched with inclusion criteria (see supplementary file 2, Table e1).

All excluded studies are characterized in the section “Excluded studies”.

#### Data extraction and management

Data extraction was done by HS and controlled by JH independently. In case of disagreement, consensus was made by discussion.

#### Assessment of study endpoints

We analyzed all endpoints from the extraction sheet and summarized them into ten categories listed in supplementary file 4 (Table e3).

## Results

The search concerning mistletoe therapy revealed 3296 hits. Of these, 1157 duplicates were removed. After title-abstract-screening 639 studies remained, including 290 clinical and 381 preclinical studies. After fulltext-screening 102 clinical publications reporting data from 108 different clinical studies remained and underwent further investigation (see supplementary file 1, Fig. e1).

### Characteristics of included studies

#### Heterogeneity of included study populations

Overall, 19.441 patients from 108 studies were included in this review (missing data on included patients in the publication of Majewski and Bentele (1963), therefore not included in the total number of included patients). Of these, 18.176 patients were analyzed. The study populations were very heterogeneous. Three of the included studies were conducted with pediatric patients (Cho and Kim 2018: *n* = 1; Seifert et al. 2007: *n* = 1; Zuzak et al. 2018: *n* = 10). Healthy subjects were investigated in three studies (Gorter et al. 1998: *n* = 7; Huber et al. 2002: *n* = 48, 2011: *n* = 71). Due to the large heterogeneity of the included patients in terms of variation in patient characteristics, comparability of the different studies among themselves is limited.

#### Great heterogeneity in study designs used for clinical trials

The included studies show a variety of different study designs (see supplementary file 5, Table e4). In one study, the study type is not specified (Gorter et al. 1998). Two of the studies are feasibility studies (Gaafar et al. 2014; Loewe-Mesch et al. 2008). The 36 cohort studies can be divided into controlled and non-controlled studies. Of these, 29 studies (16 publications respectively) are designed as controlled studies (Augustin et al. 2005; Bock et al. 2004a, 2014; Beuth et al. 2008; Friedel et al. 2009; Grossarth-Maticek and Ziegler 2006a, b, 2007a, b, 2007c, 2008; Matthes et al. 2010; Schumacher et al. 2003; Thronicke et al. 2017, 2020a; Zaenker et al. 2012) Of the studies published by Grossarth-Maticek, eight were randomized matched-pair studies, and eleven were non-randomized matched-pair studies. The remaining cohort studies are non-controlled (Brandenberger et al. 2012; Oei et al. 2019a, b; Schad et al. 2017, 2018a, b; Thronicke et al. 2020b). There is a big heterogeneity regarding study designs and thus also a divergence in terms of level of evidence and methodological quality of the included studies. Thus, an evaluation and assessment regarding the effect of mistletoe therapy becomes difficult.

#### Huge variety in cancer types investigated in clinical trials

The most common types of cancer included in this review were breast cancer, pancreatic cancer, colorectal cancer and malignant melanoma. A more detailed overview is shown in supplementary file 6 (Table e5). In three studies, healthy volunteers were recruited (Gorter et al. 1998: number of patients included (nI) = 7, number of patients analyzed (nA) = 7; Huber et al. 2002: nI = 48, nA = 48; Huber et al. 2011: nI = 71, nA = 71). In the included studies, mistletoe therapy is used in patients with a variety of different cancer types. Thus, comparability of the studies is limited.

#### Great variety in mistletoe preparations used for clinical trials

Anthroposophical or homeopathically produced mistletoe preparations (Helixor^®^, Iscador^®^, Abnobaviscum^®^, Iscucin^®^, Isorel^®^, Plenosol^®^ and Viscum mali e planta tota^®^) as well as phytotherapeutic mistletoe preparations (Eurixor^®^ and Lektinol^®^) were used in the studies. In 75 studies, only one mistletoe preparation was used. In ten studies, mistletoe preparations from different host trees were used, but mistletoe preparations had been obtained from the same pharmaceutical company. In the remaining 20 studies two or more different mistletoe preparations were tested.

In some studies, the authors only reported that patients received mistletoe extracts and the mistletoe preparation was not specified (Elsasser-Beile et al. 2005a; Hwang et al. 2019; Oh 2020; Werthmann et al. 2017a, 2018c). In another study, it was only reported that patients received mistletoe lectin (Goebell et al. 2002). Moreover, in some studies Viscum-fraxini-2^®^ was given as study medication (Ebrahim et al. 2010; El-Kolaly et al. 2016; Gaafar et al. 2014; Mabed et al. 2004). In three of these studies, the pharmaceutical company was not explicitly mentioned (Ebrahim et al. 2010; Gaafar et al. 2014; Mabed et al. 2004). Thus it was not clear from which pharmaceutical company the preparation was delivered and in the study of Gaafar et al. (2014) which dosage was used.

Supplementary file 7 (Table e6) shows the mistletoe preparations used in relation to the different types of cancer.

In two of studies with healthy volunteers, Iscador^®^ preparations such as Iscador^®^ QuFrF (*n* = 4) or Iscador^®^ Qu Spezial (*n* = 3) [*N* = 7, Gorter et al. (1998)] and Iscador^®^ Qu Spezial (*n* = 16) or Iscador^®^ P (*n* = 16) [*N* = 32, Huber et al. (2002)] were used. In the third study, Iscucin^®^ Populi and Viscum mali e planta tota^®^ which is usually used in patients with anthropathies were used as study medications [*N* = 30, Huber et al. (2011)].

A variety of different mistletoe preparations was used to treat cancer patients. Due to the heterogeneity of the mistletoe preparations used, no relationship between mistletoe preparation and type of cancer can be observed.

#### Different ways of application of mistletoe preperations

Mistletoe preparations are approved for subcutaneous application. Accordingly, in most of the studies, VAE was applied subcutaneously. In 63 studies, subcutaneous injections was the only way of application. In contrast, in 17 studies s.c. application and at least one other application form was used. Other common forms of mistletoe application were off-lable intratumoural or intravenous injections. Rare forms of application were intrapleural, intraperitoneal, intravesical, intrathecal or oral. In nine studies, the form of application was not specified. Despite the only approval of mistletoe preparations for subcutaneous applications, mistletoe preparations were applied off-label in many different other ways.

#### Great variety of dosage and dosage schedules used in clinical trials

The dosage of the mistletoe preparations varied greatly (see supplementary file 8, Table e7). In some studies, the dosage of the mistletoe preparations used was not specified. In other studies, however, only an average or median dosage was given (Bock et al. 2004a, 2014; Schläppi et al. 2017; Zaenker et al. 2012). There is a big heterogeneity regarding the dosages and dosage schedules. In many studies, the dosage schedules for subcutaneous application deviate from the recommendations of the respective manufacturers. Also, the dosage schedules used for off-label injections, for which no dosage recommendations exist, varies greatly.

#### Heterogeneity of indications for usage of mistletoe and endpoints assessed

Across the studies, outcome parameters varied considerably. All in all, the endpoints may be sorted in ten categories which are listed in supplementary file 9 (Table e8). including the respective studies. The heterogeneity of the indications of mistletoe in the studies points to a lack of defined indications and aims in mistletoe treatments.

### Excluded studies

Four double publications published in German as well as in English were excluded (Lenartz et al. 2000, 2001; Piao et al. 2004; Schierholz et al. 2003; Tröger et al. 2009; Tröger et al. 2011; Schumacher et al. 2002, 2003).

Apart from that, two other publications were excluded because they were reporting almost completely overlapping data from the same study (Bock et al. 2004a, b; Elsasser-Beile et al. 2005a, b).

Besides that, two randomized datasets based on patients with breast cancer or different cancer types were excluded (Grossarth-Maticek et al. 2001). The first dataset studying breast cancer patients was re-analyzed in a later publication which is included in this review instead (Grossarth-Maticek and Ziegler 2006b). The other randomized dataset based on various cancer types was excluded. As it is not clear to what extent the data overlap with subsequent studies (Grossarth-Maticek and Ziegler 2007b, 2007c, 2008).

Finally, another publication was excluded because it reported preliminary results from a study published later (Longhi et al. 2009, 2014).

## Discussion

We analyzed 108 clinical studies, comprising 19.441 participants including healthy volunteers and pediatric patients in three studies each. The studies comprise nine different mistletoe preparations in various dosages and eight different ways of application. In the studies by Grossarth-Maticek et al., it may not be excluded that data from control patients were used more than once, as in the articles it is said that „control patients were only used once in the mistletoe studies and were never used in other studies”, which does not exclude the same patients being used as control in several mistletoe studies.

Less than one third of the included 108 studies were randomized controlled studies showing a formally high level of evidence. However, even these studies lack methodological quality. The study of Cho et al. 2016 that was named a phase III trial but was conducted as a single-armed study including no control group. The argumentation of the authors that a control group was not possible due to a lack of a standard treatment in pleurodesis is not correct. Talkum as well as bleomycin would have been well established options (Ried and Hofmann 2013).

Another 22 of the included studies are case reports or case series with the lowest level of evidence and thus little informative value with regard to the safety and efficacy of mistletoe therapy.

Anthroposophical or homeopathically produced mistletoe preparations (Helixor^®^, Iscador^®^, Abnobaviscum^®^, Iscucin^®^, Isorel^®^, Plenosol^®^ and Viscum mali e planta tota^®^) as well as phytotherapeutic mistletoe preparations (Eurixor^®^ and Lektinol^®^) were used in the studies. They were applied subcutaneously, intravenously and intratumorally.

All in all, it can be concluded that there is a considerable heterogeneity with respect to different study populations, study types, mistletoe preparations, ways of application and dosage schedules as well as various endpoints. The application of mistletoe preparations does not seem to follow any transparently defined plan with respect to types of mistletoe, type of preparation or schedule of administration. In most studies, the positive effects in terms of survival, tumor response, symptom control, quality of life etc. were attributed to mistletoe therapy. Differences in study concepts and endpoints points to a lack of defined indications and aims in mistletoe treatments in general.

### Mistletoe preparations and host trees

A variety of different mistletoe preparations was used to treat cancer patients. Due to the heterogeneity of the mistletoe preparations used, no comparability between different studies or within single studies using different types of mistletoe preparations or host trees is possible. Moreover, no relationship between mistletoe preparation and type of cancer can be observed. This results in a severely limited comparability of studies with regard to the different cancer entities and mistletoe therapy in oncology in general.

Analyzing the methods sections of all articles, there are no information on how the selection of the respective mistletoe preparation took place. None of the articles provided any argument which type of preparation (homeopathic, anthroposophic, standardized) or which host tree was chosen due to which selection criteria. Considering preparations from different companies, funding may have been the reason of the selection. For example, all studies by Grossarth–Maticek used Iscador^®^.

In breast cancer patients for example, six different mistletoe preparations including AbnobaViscum^®^, Helixor^®^, Iscador^®^, Eurixor^®^, Iscucin^®^ and Lektinol^®^ from three different host trees (abietis, mali and pini). Yet, only in six studies information on host trees was given. In only three studies, mistletoe preparations from different pharmaceutical companies were used (Oei et al. 2018, 2019a; Pelzer et al. 2018; Tröger et al. 2009, 2012, 2014b, 2016) and moreover, in only one study, patients were treated with a mistletoe preparation from one company grown on different host trees (Beuth et al. 2008). In case of pancreatic cancer five different mistletoe preparations including AbnobaViscum^®^, Isador^®^, Helixor^®^, Eurixor^®^ and Iscucin^®^ from seven different host tress (abietis, aceris, mali, pini, fraxini, quercus and salicis) were used and in only two studies, the patients received different mistletoe preparations from different pharmaceutical companies (Schad et al. 2014; Werthmann et al. 2018b). In two studies mistletoe preparations from the same preparation type, but from different host trees were given (Thronicke et al. 2020a; Werthmann et al. 2019a).

The results are the same for colorectal cancer (three mistletoe preparations from four different host trees, only one study with mistletoe grown on different host trees), malignant melanoma (three mistletoe preparations from five host trees), lung cancer (three mistletoe preparations), renal cell carcinoma (four mistletoe preparations from four host trees, one study with different host trees) or gynecological cancer (two mistletoe preparations from three host trees).

In many studies, the exact information on the host tree is missing (Bock et al. 2004a, Elsasser-Beile et al. 2005a, Eom et al. 2017, 2018, Goebell et al. 2002, Grossarth-Maticek and Ziegler 2006a, 2006b, 2007a, 2007c, 2008, Günczler et al. 1968, Günczler and Salzer 1969, Hwang et al. 2019, Leroi 1977, Majewski and Bentele 1963, Matthes et al. 2010, Oei et al. 2018, 2019a, 2019b, Oh 2020, Schad et al. 2014, 2018a, Son et al. 2010, Thronicke et al. 2020b).

All in all, from the beginning of the studies available in the international literature, information on which type of mistletoe preparation is used for which special cancer situation is missing. Moreover, neither the results from single studies nor a cross referencing from different studies reveals any information that would allow to derive recommendations for indications.

Also in those studies using preparations from different companies or from different host trees no comparison of outcome data with respect to survival, or side effects of cancer therapy is provided (Augustin et al. 2005; Beuth et al. 2008; Brandenberger et al. 2012; Friedel et al. 2009; Kjaer 1989; Oei et al. 2018, 2019a, b; Pelzer et al. 2018; Schad et al. 2014, 2017, 2018a, b; Schläppi et al. 2017; Steele et al. 2014a; Stumpf et al. 2000, 2003; Thronicke et al. 2017, 2018, 2020a, b; Tröger et al. 2009, 2012, 2014b, 2016; Werthmann et al. 2014, 2017a, b, 2018b, c, 2019a) with the exception of Huber et al. (2002, 2011) and Steele et al. (2014b, 2015). Only four studies directly compared at least two different mistletoe preparations regarding safety and efficacy (Huber et al. 2002, 2011; Steele et al. 2014b, 2015). Yet, two of these studies were conducted with healthy subjects and analyzed immunological parameters The studies of Steele et al. include a direct comparison between mistletoe preparations from different pharmaceutical companies and different host trees with respect to the occurrence of adverse drug reactions.

In a three-armed randomized study of Pelzer et al. (2018) safety, impact on disease-free-survival and clinical response of mistletoe in breast cancer patients were evaluated. In the evaluation, the two groups receiving mistletoe preparations were summarized into one group. In fact the same applies to other studies including a three-armed study of Tröger et al. (2012, 2014b, 2016,2009, ), in which different mistletoe preparations from different pharmaceutical companies or different host trees were given.

### Dosage of mistletoe

Dosage or dosage regimens varied strongly in the studies. Due to the heterogeneity of dosage and dosage regimens within studies and between studies of the endpoints the comparability of the different studies is severely limited.

While there are some studies providing no data at all, in case of breast cancer for example, eight different dosages or different dosage regimens were used, for colorectal cancer there are four different dosages or dosage schedules and for lung cancer five different dosages or dosage regimens. Also for dosage, information on how the dosage was selected is missing in the methods or results sections. Moreover, in some studies, mistletoe was applied in schedules with increasing dosage and/or pausing for several days ranging from one week (Wode et al. 2009) to three months (Goebell et al. 2002).

There are only a few studies comparing the different dosages or dosage schedules of mistletoe preparations (Huber et al. 2017; Rose et al. 2015; Schad et al. 2017; Semiglasov et al. 2004; Steele et al. 2014a, b).

The objective of the study of Semiglasov et al. (2004) was to investigate the safety and impact of Lektinol^®^ on quality of life, chemotherapy related side effects and immunological parameters. Lektinol^®^ at concentrations of 10, 30 or 70 ng mistletoe lectin per ml were used. The analysis of GLQ-8 sum and Spitzer’s uniscale resulted in statistically significant effects on quality of life by using the medium dose of Lektinol^®^. However, no significant differences in favor of the low and high dose could be established. In contrast, no relevant differences in quality of life between treatment groups could be detected measured by the approved EORTC-QLQ-C30 scale. As a phase I study is lacking on any of the mistletoe preparations, systematic information on dosage is missing.

### Duration of treatment

Duration of mistletoe treatment varied strongly in the studies ranging from a single dose given on one day to the application of mistletoe preparations for several years. Moreover, the duration of treatment frequently varied within the studies. In these cases, data on mean or median durations partly with data on range of mistletoe treatment were provided (Augustin et al. 2005; Bar-Sela and Haim 2004; Bock et al. 2004a, 2014; Cho et al. 2016; Eom et al. 2018; Friedel et al. 2009; Grossarth-Maticek and Ziegler 2006b, 2007a, 2008; Mabed et al. 2004; Matthes et al. 2010; Oei et al. 2019b; Schad et al. 2018a; Schumacher et al. 2003; Steele et al. 2014a; Stumpf et al. 2000, 2003; Thronicke et al. 2017, 2018; Zaenker et al. 2012; Zuzak et al. 2018). In some studies, patients were treated with mistletoe preparations until death (Friess et al. 1996; Kjaer 1989) or till unacceptable toxicity/ tumor progression occurred or the patient chose to discontinue treatment (Brinkmann and Hertle 2004; Ebrahim et al. 2010; Kleeberg et al. 2004).

Yet, defined durations of treatment or transparent criteria for a decision to stop mistletoe treatment (apart from tumor progression, tumor response, adverse events and private reasons) are mostly lacking. Moreover, in several studies no data on treatment duration was provided (Bar-Sela et al. 2006; Beuth et al. 2008; Cazacu et al. 2003; El-Kolaly et al. 2016; Elsasser-Beile et al. 2005a; Grossarth-Maticek and Ziegler 2006a, 2007b, c; Günczler et al. 1968; Majewski and Bentele 1963; Oei et al. 2019a; Piao et al. 2004; Schad et al. 2014, 2017, 2018b; Schläppi et al. 2017; Seifert et al. 2007; Shaw et al. 2004; Thronicke et al. 2020a).

### Comparison of mistletoe with placebo or another active treatment

As mistletoe is well-known by patients and physicians in German speaking countries and the procedure of regular injections, attention by the physician and the patient may induce a strong placebo effect. As patients may show a local immunological reaction, blinding is difficult.

Four studies were conducted as placebo-controlled studies (Huber et al. 2002, 2011; Semiglasov et al. 2004; Semiglazov et al. 2006).

The analysis of Huber et al. (2002) shows that local reactions occurred, regardless of whether they received Iscador^®^ P, Q or placebo. However, local reactions were significantly less frequent in the placebo group. Furthermore, mean subjective tolerability towards the injection of Iscador^®^ P, Q, and placebo was significantly lower in the patients treated with mistletoe preparation. Moreover, eosinophil rate was increased significantly in the patients treated with Iscador^®^ Q, probably related to its high content of lectins. Yet. the authors point to the fact that no deblinding of the assessors happened.

The same concerns on deblinding apply to the study by Semiglasov et al. (2004) and Semiglazov et al. (2006).

### Application

In the studies, mistletoe preparations were administered by different ways of application. Most frequently, the probands received mistletoe preparations subcutaneously, as recommended by the manufacturers. The second most common way was intravenous administration of mistletoe preparations. According to the respective manufacturer, this type of application is only recommended for Lektinol^®^ and Eurixor^®^. The other preparations are given as off-label intravenous applications. Accordingly, no dosage recommendations from the respective manufacturer are available. Only in two studies the dose schedules are mentioned: according to the classical phase I 3 + 3 dose escalation schedule (Huber et al. 2017) or in ratio to the body surface area (Zuzak et al. 2018).

### Indication for usage of mistletoe

Across the studies, efficacy is assessed with respect to survival as well as quality of life. From this, it remains unclear whether mistletoe treatment is considered a tumor therapy or a treatment of side effects.

It is most difficult to compare and integrate data of different studies as the both survival as well as quality of life are assessed using different time points and instruments respectively.

For example, in terms of survival, overall survival/ tumor-related survival, disease-free-survival, postrelapse-disease-free-survival, relapses und metastases, recurrence rate, tumor progression/ time-to-tumor-progression, progression-free-survival, tumor response/ tumor remission have been reported.

Improvement if quality of life of patients receiving mistletoe preparations is an aim of mistletoe therapy. As Horneber et al. (2008) already reported, there is a big heterogeneity between the included studies. First of all, quality of life was assessed in different ways using 13 different multidimensional and unidimensional questionnaires. In two studies the patients were additionally interviewed (Brandenberger et al. 2012; Reynel et al. 2020). Due to the fact, that EORTC-QLQ and FACT-G are the only established QoL instruments in cancer research, the informative value of the studies using other QoL instruments is limited. Furthermore, not all of the studies assessing quality of life are designed as controlled studies, making it not able to compare the effect of mistletoe therapy regarding quality of life with a control group (Brandenberger et al. 2012; Friess et al. 1996; Kjaer 1989). Second, in some studies a selection bias is possible due to inhomogenities of baseline uality of life data (Kim et al. 2012; Klose et al. 2003; Semiglasov et al. 2004; Semiglazov et al. 2006). In other studies there is missing information whether study and control groups are comparable in terms of quality of life at baseline (Enesel et al. 2005; Piao et al. 2004).

To summarize, due to the heterogeneity of the endpoints assessing efficacy mistletoe therapy the comparability of the different studies in limited and scientists conducting systematic review and more so meta-analyses face severe problems in data syntheses.

## Limitations of this work

There are several limitations of this review. First of all, there is a big heterogeneity of studies included. On the one hand, the strength lies in a broad overview of studies on mistletoe treatment. On the other hand, the power of this review is limited due to a lack of evidence and methodological quality in the majority of the included studies. Moreover, it was not possible to perform a meta-analysis because of the heterogeneity of included studies. Only a descriptive overview of the studies could be conducted. Second, the interpretation of data was limited due to the high heterogeneity of patients, endpoints and mistletoe extracts in different dosages and ways of application. Third, only articles published in German or English were included.

## Conclusion

Despite a large number of clinical studies and reports, there is a complete lack of transparently reported, structured procedures considering all fields of mistletoe therapy. This applies to type of mistletoe extract, host tree, preparation, treatment schedules as well as indication with respect of type of cancer and the respective treatment aim. All in all, despite several decades of clinical mistletoe research, no clear concept of usage is discernible and, from an evidence-based point of view, there are serious concerns on the scientific base of this part of anthroposophical treatment.

## Supplementary Information

Below is the link to the electronic supplementary material.

Supplementary file1 (DOCX 928 KB)

Supplementary file2 (DOCX 20 KB)

Supplementary file3 (DOCX 24 KB)

Supplementary file4 (DOCX 19 KB)

Supplementary file5 (DOCX 146 KB)

Supplementary file6 (DOCX 164 KB)

Supplementary file7 (DOCX 173 KB)

Supplementary file8 (DOCX 213 KB)

Supplementary file9 (DOCX 332 KB)

## Data Availability

All data generated or analysed during this study are included in this published article [and its supplementary information files].
