# Supplementary material for: Systematic analysis of mistletoe prescriptions in clinical studies
Source: J Cancer Res Clin Oncol. 2022 Dec 9;149(9):5559–71. doi: 10.1007/s00432-022-04511-2 (PMC10356894; doi:10.1007/s00432-022-04511-2)

**Systematic analysis of mistletoe prescripitions in clinical studies**

Henrike Staupe^1^, Judith Buentzel^2^, Christian Keinki^1^, Jens Buentzel^3^, Jutta Huebner^1^

^1^ Klinik für Innere Medizin II; Hämatologie und Onkologie, Universitätsklinikum Jena

^2^Klinik für Hämatologie und medizinische Onkologie, Universitätsmedizin Göttingen

^3^Klinik für HNO-Erkrankungen, Südharz-Klinikum Nordhausen

Corresponding author: Henrike Staupe. h.staupe@web.de

Journal: Journal of cancer research and clinical oncology

**Fig. e1** Consort diagram


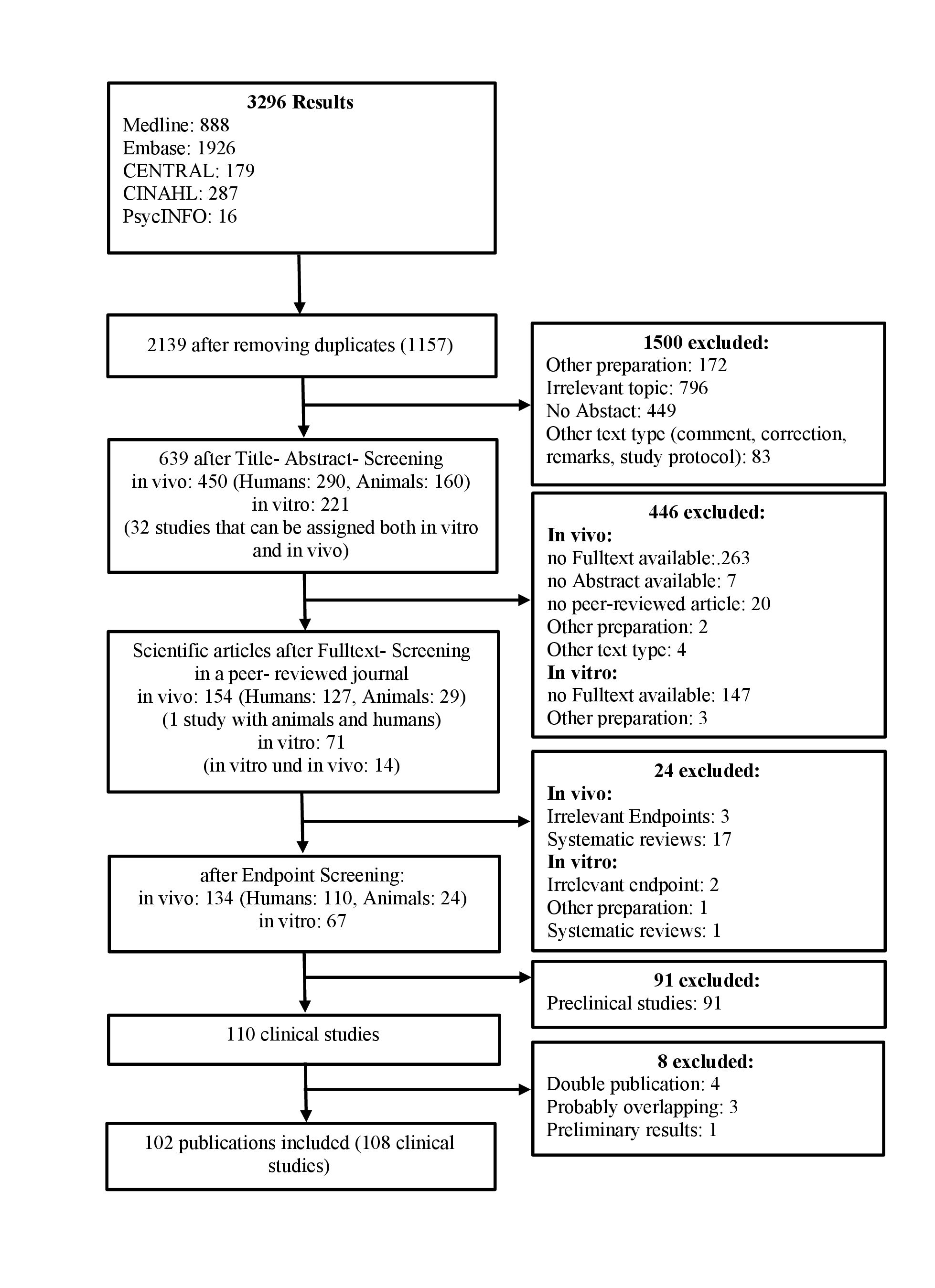

Supplement: Supplementary file 1 — Supplementary file1 (DOCX 928 KB) [file 432_2022_4511_MOESM1_ESM.docx]
