# Supplementary material for: Systematic analysis of mistletoe prescriptions in clinical studies
Source: J Cancer Res Clin Oncol. 2022 Dec 9;149(9):5559–71. doi: 10.1007/s00432-022-04511-2 (PMC10356894; doi:10.1007/s00432-022-04511-2)
Supplement: Supplementary file 2 — Supplementary file2 (DOCX 20 KB) [file 432_2022_4511_MOESM2_ESM.docx]

**Systematic analysis of mistletoe prescripitions in clinical studies**

Henrike Staupe^1^, Judith Buentzel^2^, Christian Keinki^1^, Jens Buentzel^3^, Jutta Huebner^1^

^1^ Klinik für Innere Medizin II; Hämatologie und Onkologie, Universitätsklinikum Jena

^2^Klinik für Hämatologie und medizinische Onkologie, Universitätsmedizin Göttingen

^3^Klinik für HNO-Erkrankungen, Südharz-Klinikum Nordhausen

Corresponding author: Henrike Staupe. h.staupe@web.de

Journal: Journal of cancer research and clinical oncology

**Table e1** Inclusion and exclusion criteria

|  | Inclusion criteria | Exclusion criteria |
| --- | --- | --- |
| Subjects | Cancer Patients (all entities and stages),  Patients with carcinoma in situ,  Healthy volunteers,  Adult (age > 18) and pediatric patients (age < 18) | Primary prevention,  Preclinical studies |
| Intervention | Every intervention with mistletoe extract  No restrictions regarding mistletoe extract, dose, mode of application |  |
| Study design | All types of clinical studies | Preclinical studies |
| Type of publication | Original studies,  Scientific articles published as full papers in a peer-reviewed journal | Reviews, Editorials, Letters, Comments, Corrections, Study protocols |
| Outcome | Viscum album extract related toxicity/ adverse events/ side effects/ adverse drug reactions, Quality of life, Psychosomatic self- regulation, Sense of coherence (Inner coherence and resilience + hermos coherence), Tumor response, Tumor remission, Overall Survival/ Tumor-related survival, Disease-free-survival, Postrelapse-disease-free-survival, Relapses und metastases, Recurrence rate, Tumor progression/ Time-to-tumor-progression, Progression-free-survival, Immunological parameters including Neuropenia and Body temperature, Cost outcomes and Cost-effectiveness, Effect on side effects of conventional cancer therapy including body weight and cancer related fatigue and on disease related symptoms, Molecular activity of tumor reduction, Impact on pleural effusion and aszites |  |
| Others | Language: German or English  Scientific articles published as full papers in a peer-reviewed journal | Abstracts, other languages |
