# Supplementary material for: Systematic analysis of mistletoe prescriptions in clinical studies
Source: J Cancer Res Clin Oncol. 2022 Dec 9;149(9):5559–71. doi: 10.1007/s00432-022-04511-2 (PMC10356894; doi:10.1007/s00432-022-04511-2)
Supplement: Supplementary file 3 — Supplementary file3 (DOCX 24 KB) [file 432_2022_4511_MOESM3_ESM.docx]

**Systematic analysis of mistletoe prescripitions in clinical studies**

Henrike Staupe^1^, Judith Buentzel^2^, Christian Keinki^1^, Jens Buentzel^3^, Jutta Huebner^1^

^1^ Klinik für Innere Medizin II; Hämatologie und Onkologie, Universitätsklinikum Jena

^2^Klinik für Hämatologie und medizinische Onkologie, Universitätsmedizin Göttingen

^3^Klinik für HNO-Erkrankungen, Südharz-Klinikum Nordhausen

Corresponding author: Henrike Staupe. h.staupe@web.de

Journal: Journal of cancer research and clinical oncology

**Table e2** Search strategy for each database (Freuding et al. 2019a, b)

|  | Search strategy |
| --- | --- |
| Medline/ Embase via Ovid | 1. mistletoe$.mp. or exp Mistletoe/ or viscum album.mp. or exp Viscum album/ or (“ABNOBAViscum” or “Lektinol” or “Plenosol” or “Isorel” or “Iscucin” or “Iscador” or “Iscar” or “Helixor” or “Eurixor” or “Vysorel”).mp  2. exp neoplasms/ or neoplasm$.mp. or cancer$.mp. or tumo?r$.mp. or malignan$.mp. or oncolog$.mp. or carcinom$.mp. or leuk?emia.mp. or lymphoma.mp. or sarcom$.mp  3. 1 and 2  4. limit 3 to English or limit 3 to German |
| CENTRAL | #1. [mh mistletoe] or mistletoe? or “Viscum album” or “Viscum” or “ABNOBAViscum” or “Lektinol” or “Plenosol”  or “Isorel” or “Iscucin” or “Iscador” or “Iscar” or “Helixor” or “Eurixor” or “Vysorel”  #2. [mh neoplasms] or neoplasm* or cancer? or tum*r? or malignan* or oncolog* or carcinom* or leuk*mia or  “lymphoma” or sarcoma?  #3. #1 and #2 |
| CINAHL | S1. (MH “Mistletoe” OR TX Mistletoe OR TX “Viscum album” OR TX “Viscum” OR TX “ABNOBAViscum” OR  TX “Lektinol” OR TX “Plenosol” OR TX “Isorel” OR TX “Iscucin” OR TX “Iscador” OR TX “Iscar” OR TX  “Helixor” OR TX “Eurixor” OR TX “Vysorel”)  S2. MH “Neoplasms+” OR TX neoplasm* OR TX cancer OR TX tumo#r OR TX malignan* OR TX oncolog* OR  TX carcinom* OR TX leuk#emia OR TX lymphoma OR TX sarcoma  S3. (LA German OR LA English)  S4. S1 AND S2 AND S3 |
| PsycINFO | S1. (TX Mistletoe OR TX “Viscum album” OR TX “Viscum” OR TX “ABNOBAViscum” OR TX “Lektinol” OR TX “Plenosol” OR TX “Isorel” OR TX “Iscucin” OR TX “Iscador” OR TX “Iscar” OR TX “Helixor” OR TX “Eurixor” OR TX “Vysorel”) S2. ((DE “Neoplasms” OR DE “Benign Neoplasms” OR DE “Breast Neoplasms” OR DE “Endocrine Neoplasms” OR DE “Leukemias” OR DE “Melanoma” OR DE “Metastasis” OR DE “Nervous System Neoplasms” OR DE “Terminal Cancer”) OR (TX neoplasm* OR TX cancer OR TX tumo#r OR TX malignan* OR DE “oncology” OR TX oncolog* OR TX carcinom* OR TX leuk#emia OR TX lymphoma OR TX sarcoma) S3. (LA German OR LA English) S4. S1 AND S2 AND S3 |
| Science Citation Index  Expanded (Web of  Science) | #1. (TS=Mistletoe* OR TS="Viscum album” OR TS=Viscum OR TS="ABNOBAViscum” OR TS="Lektinol”  OR TS="Plenosol” OR TS="Isorel” OR TS="Iscucin” OR TS="Iscador” OR TS="Iscar” OR TS="Helixor” OR  TS="Eurixor” OR TS="Vysorel”)  #2. (TS=neoplasm* OR TS=cancer OR TS=cancers OR TS=>tumo$r OR TS=tumo$rs OR TS=malignan* OR  TS=oncolog* OR TS=carcinom* OR TS=leuk$emia OR TS=lymphoma OR TS=sarcoma OR TS=sarcomas)  #3. #1 AND #2 |

The numbers of the references refer to the reference list in the main manuscript.
