# Supplementary material for: Systematic analysis of mistletoe prescriptions in clinical studies
Source: J Cancer Res Clin Oncol. 2022 Dec 9;149(9):5559–71. doi: 10.1007/s00432-022-04511-2 (PMC10356894; doi:10.1007/s00432-022-04511-2)
Supplement: Supplementary file 4 — Supplementary file4 (DOCX 19 KB) [file 432_2022_4511_MOESM4_ESM.docx]

**Systematic analysis of mistletoe prescripitions in clinical studies**

Henrike Staupe^1^, Judith Buentzel^2^, Christian Keinki^1^, Jens Buentzel^3^, Jutta Huebner^1^

^1^ Klinik für Innere Medizin II; Hämatologie und Onkologie, Universitätsklinikum Jena

^2^Klinik für Hämatologie und medizinische Onkologie, Universitätsmedizin Göttingen

^3^Klinik für HNO-Erkrankungen, Südharz-Klinikum Nordhausen

Corresponding author: Henrike Staupe. h.staupe@web.de

Journal: Journal of cancer research and clinical oncology

**Table e3** Categorized endpoints

| 1 | VAE related toxicity/ adverse events/ side effects/ adverse drug reactions |
| --- | --- |
| 2 | Quality of Life, Psychosomatic self- regulation, Sense of coherence (Inner coherence and resilience + thermo coherence) |
| 3 | Tumor response, Tumor remission |
| 4 | Overall Survival/ Tumor-related survival |
| 5 | Disease-free-survival (DFS), Postrelapse-disease-free-survival (PRDFS), Relapses und metastases, Recurrence rate, Tumor progression/ Time-to-tumor-progression, Progression-free-survival (PFS) |
| 6 | Immunological parameters including neutropenia and body weight |
| 7 | Cost outcomes and Cost-effectiveness |
| 8 | Effect on side effects of conventional cancer therapy including body weight and cancer related fatigue and on disease related symptoms |
| 9 | Changes in histological and molecular characteristics in tumor tissue/tumor cells |
| 10 | Impact on pleural effusion and aszites |
