# Supplementary material for: Systematic analysis of mistletoe prescriptions in clinical studies
Source: J Cancer Res Clin Oncol. 2022 Dec 9;149(9):5559–71. doi: 10.1007/s00432-022-04511-2 (PMC10356894; doi:10.1007/s00432-022-04511-2)
Supplement: Supplementary file 5 — Supplementary file5 (DOCX 146 KB) [file 432_2022_4511_MOESM5_ESM.docx]

**Systematic analysis of mistletoe prescripitions in clinical studies**

Henrike Staupe^1^, Judith Buentzel^2^, Christian Keinki^1^, Jens Buentzel^3^, Jutta Huebner^1^

^1^ Klinik für Innere Medizin II; Hämatologie und Onkologie, Universitätsklinikum Jena

^2^Klinik für Hämatologie und medizinische Onkologie, Universitätsmedizin Göttingen

^3^Klinik für HNO-Erkrankungen, Südharz-Klinikum Nordhausen

Corresponding author: Henrike Staupe. h.staupe@web.de

Journal: Journal of cancer research and clinical oncology

**Table e4** Study design

| Study design | Number (n) |
| --- | --- |
| Randomized controlled study | 29 publications (Bar-Sela et al. 2013; Brinkmann and Hertle 2004; Cazacu et al. 2003; El-Kolaly et al. 2016; Enesel et al. 2005; Gaafar et al. 2014; Goebell et al. 2002; Huber et al. 2002, 2011; Kim et al. 2012; Kleeberg et al. 2004; Klose et al. 2003; Lenartz et al. 2000; Longhi et al. 2014, 2020; Pelzer et al. 2018; Piao et al. 2004; Schink et al. 2007; Semiglasov et al. 2004; Semiglazov et al. 2006; Son et al. 2010; Steuer-Vogt et al. 2001, 2006; Tröger et al. 2009, 2012, 2013, 2014a, b, 2016): 22 studies |
| Cohort study | 23 publications (Augustin et al. 2005; Beuth et al. 2008; Bock et al. 2004a, 2014; Brandenberger et al. 2012; Friedel et al. 2009; Grossarth-Maticek and Ziegler 2006a, b, 2007a, b, c, 2008; Matthes et al. 2010; Oei et al. 2019a, b; Schad et al. 2017, 2018a, b; Schumacher et al. 2003; Thronicke et al. 2017, 2020a, b; Zaenker et al. 2012): 36 studies |
| Case report | 19 publications (Cho and Kim 2018; Gutsch et al. 2018; Hwang et al. 2019; Oh 2020; Reynel et al. 2018, 2019, 2020; Seifert et al. 2007; Shaw et al. 2004; Werthmann et al. 2014, 2017a, b, 2018a, b, c, d, 2019a, b; Wode et al. 2009): 19 studies |
| Retrospective analysis | 6 publications (Eom et al. 2018; Lee et al. 2019; Schad et al. 2014; Schläppi et al. 2017; Stumpf et al. 2000, 2003): 6 studies |
| Observational study | 5 publications (Oei et al. 2018; Steele et al. 2014a, b, 2015; Thronicke et al. 2018): 5 studies |
| Case series | 3 publications (Eom et al. 2017; Gardin 2009; Zuzak et al. 2018): 3 studies |
| Non-randomized controlled study | 6 publications (Fellmer 1968; Günczler et al. 1968; Günczler and Salzer 1969^a;^ Leroi 1977; Loewe-Mesch et al. 2008; Majewski and Bentele 1963): 6 studies |
| Phase I Study | 1 publication (Huber et al. 2017): 1 study |
| Phase I/II Study | 3 publications (Elsasser-Beile et al. 2005a; Friess et al. 1996; Rose et al. 2015): 3 studies |
| Phase II Study | 5 publications (Bar-Sela and Haim 2004; Bar-Sela et al. 2006; Ebrahim et al. 2010; Kjaer 1989; Mabed et al. 2004): 5 studies |
| Single-arm Phase III Study | 1 publication (Cho et al. 2016): 1 study |
| No specification | 1 publication (Gorter et al. 1998): 1 study |

^a^ historical control group

The numbers of the references refer to the reference list in the main manuscript.
