# Supplementary material for: Systematic analysis of mistletoe prescriptions in clinical studies
Source: J Cancer Res Clin Oncol. 2022 Dec 9;149(9):5559–71. doi: 10.1007/s00432-022-04511-2 (PMC10356894; doi:10.1007/s00432-022-04511-2)
Supplement: Supplementary file 6 — Supplementary file6 (DOCX 164 KB) [file 432_2022_4511_MOESM6_ESM.docx]

**Systematic analysis of mistletoe prescripitions in clinical studies**

Henrike Staupe^1^, Judith Buentzel^2^, Christian Keinki^1^, Jens Buentzel^3^, Jutta Huebner^1^

^1^ Klinik für Innere Medizin II; Hämatologie und Onkologie, Universitätsklinikum Jena

^2^Klinik für Hämatologie und medizinische Onkologie, Universitätsmedizin Göttingen

^3^Klinik für HNO-Erkrankungen, Südharz-Klinikum Nordhausen

Corresponding author: Henrike Staupe. h.staupe@web.de

Journal: Journal of cancer research and clinical oncology

**Table e5** Cancer type/ Primary tumour

| Cancer type/ Primary tumour | Number (nI= Number of patients included, nA= Number of patients analyzed) |
| --- | --- |
| Breast cancer | 20 publications  Beuth et al. (2008): nI=741, nA=681;  Bock et al. (2004a): nI=1442, nA=1442;  Grossarth-Maticek and Ziegler (2006a): randomized matched-pair study (without lymphatic or distant metastases): nI=118, nA=76; non-randomized matched-pair study (without lymphatic or distant metastases): nI=210, nA=168: total nI=328, nA=244;  Grossarth-Maticek and Ziegler (2006b): randomized matched-pair study (with only lymphatic metastases): nI=34, nA=34; non-randomized matched-pair study (with local recurrences): nI=100, nA=84; non-randomized matched-pair study (with only lymphatic metastases): nI=128, nA=110; non-randomized matched-pair study (with distant metastases): nI=180, nA=166: total nI=442, nA=394;  Günczler and Salzer (1969): nI= 410, nA= 410;  Leroi (1977): nI=572, nA=547;  Loewe-Mesch et al. (2008): nI=82, nA=66;  Oei et al. (2018): nI=64, nA=64;  Oei et al. (2019a): nI=72, nA=72 (Subgroup analysis: nA=45);  Pelzer et al. (2018): nI=95, nA=89/ (Follow-up analysis: nA=85);  Schumacher et al. (2003): nI=1248, nA=689;  Semiglasov et al. (2004): nI= 272, nA=261;  Semiglazov et al. (2006): nI=352, nA=337;  Shaw et al. (2004): nI=1, nA=1;  Son et al. (2010): nI=20, nA=20  Tröger et al. (2009, 2012, 2014b, 2016) nI=95, nA= 89 (Follow-up anaylsis: nA=85);  Wode et al. (2009): nI=1, nA=1  21 studies 🡪 nI=6237, nA=5407 |
| Pancreatic cancer | 8 publications  Friess et al. (1996): nI=16, nA=16;  Matthes et al. (2010): nI=396, nA=396;  Schad et al. (2014): nI=39, nA=39;  Thronicke et al. (2020a): nI=88, nA=88;  Tröger et al. (2013, 2014a): nI=220, nA= 220 (Follow-up analysis: nA=168);  Werthmann et al. (2018b): nI=1, nA=1;  Werthmann et al. (2019a): nI=1, nA=1  7 studies 🡪 nI=761, nA=761 |
| Colorectal cancer | 6 publications  Bar-Sela and Haim (2004): nI=25, nA=25;  Bock et al. (2014): nI=324, nA=324;  Cazacu et al. (2003): nI=64, nA=64;  Friedel et al. (2009): nI=804, nA=804;  Schink et al. (2007): nI=32, nA=22;  Zaenker et al. (2012): nI=318, nA=318  6 studies 🡪 nI=1567, nA=1557 |
| Malignant melanoma | 5 publications  Augustin et al. (2005): nI=783, nA=686;  Grossarth-Maticek and Ziegler (2007a): randomized matched-pair-study (without relapses and without metastases): nI=44, nA=44; non-randomized matched-pair-study (without relapses and without metastases): nI=80, nA=64: total nI=124, nA=108;  Kleeberg et al. (2004): nI=204, nA=204;  Stumpf et al. (2003): nI=284, nA=94;  Werthmann et al. (2017a): nI=1, nA=1  6 studies 🡪 nI=1396, nA=1093 |
| Lung cancer | 4 publications  Bar-Sela et al. (2013): nI=72, nA=72;  Lee et al. (2019): nI=52, nA=52;  Schad et al. (2018b): nI=158, nA=158;  Thronicke et al. (2020b): nI=275, nA=275 (Subgroup analysis: nA=110)  4 studies 🡪 nI=557, nA=557 |
| Renal cell carcinoma | 4 publications  Brinkmann and Hertle (2004): nI=74, nA=74;  Kjaer (1989): nI=14, nA=14;  Reynel et al. (2019): nI=1, nA=1;  Werthmann et al. (2019b): nI=1, nA=1  4 studies 🡪 nI=90, nA= 90 |
| Bladder cancer | 3 publications  Elsasser-Beile et al. (2005a): nI=30, nA=30;  Goebell et al. (2002): nI=45, nA=44;  Rose et al. (2015): nI=37, nA=36  3 studies 🡪 nI=112, nA=110 |
| Ovarian cancer | 3 publications  Grossarth-Maticek and Ziegler (2007c): randomized matched-pair study (without distant metastases): nI=50, nA=42; randomized matched-pair study (with distant metastases): nI=48, nA=40; non-randomized matched-pairs study (without distant metastases): nI=198, nA=150; non-randomized matched-pair study (with distant metastases): nI=132, nA=124: total nI=428, nA=356;  Hwang et al. (2019): nI=1, nA=1;  Werthmann et al. (2018c): nI=1, nA=1  6 studies 🡪 nI=430, nA=358 |
| Osteosarcoma | 2 publications  Longhi et al. (2014, 2020): nI=20, nA= 20  1 study 🡪 nI=20, nA=20 |
| Cervical cancer | 3 publications  Fellmer (1968): nI= 790, nA= 790;  Grossarth-Maticek and Ziegler (2007b): randomized matched-pair study: nI= 38, nA=38; non-randomized matched-pair study (without distant metastases): nI=212, nA= 204; randomized matched-pair study (with distant metastases): nI= 140, nA=132: total nI=390, nA=374;  Reynel et al. (2018): nI=1, nA=1;  5 studies 🡪 nI=1181, nA=1165 |
| Glioma | 1 publication  Lenartz et al. (2000): nI=38, nA=38  1 study 🡪 nI=38, nA=38 |
| Gastric cancer | 3 publications  Günczler et al. (1968): nI=186, nA= 168;  Kim et al. (2012): nI=32, nA=29;  Oh (2020): nI=1, nA=1  3 studies 🡪 nI=219, nA=198 |
| Liver cancer | 2 publications  Ebrahim et al. (2010): nI=120, nA=120;  Mabed et al. (2004): nI=23, nA=23  2 studies 🡪 nI=143, nA=143 |
| Head and neck cancer | 2 publications  Steuer-Vogt et al. (2001, 2006): nI=477, nA=477 (Follow-up anaylsis: nA=399)  1 study 🡪 nI=477, nA=477 |
| Diffuse large B-cell lymphoma (DLBCL) | 1 publication  Gutsch et al. (2018): nI=1, nA=1  1 study 🡪 nI=1, nA=1 |
| Thymic neuroendocrine tumor (TNET) | 1 publication  Reynel et al. (2020): nI=1, nA=1  1 study 🡪 nI=1, nA=1 |
| Rectal carcinoma | 1 publication  Werthmann et al. (2018a): nI=1, nA=1  1 study 🡪 nI=1, nA=1 |
| Merkel cell carcinoma | 1 publication  Werthmann et al. (2018d): nI=1, nA=1  1 study 🡪 nI=1, nA=1 |
| Adenoid cystic carcinoma | 1 publication  Werthmann et al. (2014): nI=1, nA=1  1 study 🡪 nI=1, nA=1 |
| Mesothelioma | 1 publication  Werthmann et al. (2017b): nI=1, nA=1  1 study 🡪 nI=1, nA=1 |
| Corpus uteri cancer | 1 publication  Grossarth-Maticek and Ziegler (2008): randomized matched-pair (without distant metastases): nI=76, nA=60; randomized matched-pair (with distant metastases): nI=52, nA=52; non-randomized matched pair study (without distant metastases): nI=282, nA=206; non-randomized matched pair study (with distant metastases): nI=200, nA=190: total nI=610, nA=508  4 studies 🡪 nI=610, nA=508 |
| Hematological and lymphatic cancers | 1 publication  Stumpf et al. (2000): nI=237, nA=237  1 study 🡪 nI=237, nA=237 |
| Histiocytosis | 1 publication  Seifert et al. (2007): nI=1, nA=1  1 study 🡪 nI=1, nA=1 |
| Malignant pleural effusion | 1 publication  Cho and Kim (2018): nI=1, nA=1  1 study 🡪 nI=1, nA=1 |
| Various cancer types | 23 publications  Bar-Sela et al. (2006): nI=25, nA=23;  Brandenberger et al. (2012): nI=25, nA=12;  Cho et al. (2016): nI=68, nA=62;  El-Kolaly et al. (2016): nI=45, nA=45;  Enesel et al. (2005): nI=70, nA=70;  Eom et al. (2017): nI=3, nA=3  Eom et al. (2018): nI=43, nA=43;  Gaafar et al. (2014): nI=23, nA=23;  Gardin (2009): nI=4, nA=4;  Huber et al. (2017): nI=21, nA=21;  Klose et al. (2003): nI=233, nA=224;  Majewski and Bentele (1963): nI= not clear, nA=124;  Oei et al. (2019b): nI=106, nA=106 (Subgroup analysis: nA=30);  Piao et al. (2004): nI=233, nA=224;  Schad et al. (2017): nI=1361, nA=1361;  Schad et al. (2018a): nI=56, nA=56;  Schläppi et al. (2017): nI=59, nA=59;  Steele et al. (2014a): nI=1923, nA=1923;  Steele et al. (2014b): nI=475, nA=475;  Steele et al. (2015): nI=123, nA=123;  Thronicke et al. (2017): nI=16, nA=16;  Thronicke et al. (2018): nI=310, nA=310;  Zuzak et al. (2018): nI=10, nA=10;  22 studies 🡪 nI=5232, nA=5326 |

nI: Number of patients included, nA: Number of patients analyzed

The numbers of the references refer to the reference list in the main manuscript.
