# Supplementary material for: Systematic analysis of mistletoe prescriptions in clinical studies
Source: J Cancer Res Clin Oncol. 2022 Dec 9;149(9):5559–71. doi: 10.1007/s00432-022-04511-2 (PMC10356894; doi:10.1007/s00432-022-04511-2)
Supplement: Supplementary file 7 — Supplementary file7 (DOCX 173 KB) [file 432_2022_4511_MOESM7_ESM.docx]

**Systematic analysis of mistletoe prescripitions in clinical studies**

Henrike Staupe^1^, Judith Buentzel^2^, Christian Keinki^1^, Jens Buentzel^3^, Jutta Huebner^1^

^1^ Klinik für Innere Medizin II; Hämatologie und Onkologie, Universitätsklinikum Jena

^2^Klinik für Hämatologie und medizinische Onkologie, Universitätsmedizin Göttingen

^3^Klinik für HNO-Erkrankungen, Südharz-Klinikum Nordhausen

Corresponding author: Henrike Staupe. h.staupe@web.de

Journal: Journal of cancer research and clinical oncology

**Table e6** Mistletoe preparations in relation to different types of cancer

| Cancer type | Mistletoe preparation |
| --- | --- |
| Breast cancer | Helixor^®^ M/ A/ P: N=167 (Beuth et al. 2008);  Iscador^®^ (n=710): N=710 (Bock et al. 2004a);  Iscador^®^ (n=122): N=122 🡪 one randomized study: N=38, one non-randomized study: N=84 (Grossarth-Maticek and Ziegler 2006a);  Iscador^®^ (n=197): N=197 🡪 one randomized study: N=17, three non-randomized studies: N=42, N=55, N=83 (Grossarth-Maticek and Ziegler 2006b);  Iscador^®^ (n=257): N=257 (Günczler and Salzer 1969);  Iscador^®^ (n=319): N=319 (Leroi 1977);  Iscador^®^ M Spezial (n=33): N=33 (Loewe-Mesch et al. 2008);  AbnobaViscum^®^ (n=24), Iscador^®^ (n=8), Helixor^®^ (n=14), Iscucin^®^ (n=4): N=39 (Oei et al. 2018);  AbnobaViscum^®^ (n=8), Helixor^®^ (n=4), Iscador^®^ (n=6), Iscucin^®^ (n=2): N= 16 (Oei et al. 2019a);  Helixor^®^ A (n=34), Iscador^®^ M Spezial (n=30): N= 64 (Pelzer et al. 2018);  Eurixor^®^ (n=219): N=219 (Schumacher et al. 2003);  Lektinol^®^ (n=202, analyzed: 195): N=202, analyzed: 195 (Semiglasov et al. 2004);  Lektinol^®^ (n=176, analyzed: 169): N=176, analyzed: 169 (Semiglazov et al. 2006)  Iscador^®^ M (n=1): N=1 (Shaw et al. 2004);  Helixor^®^ (n=10): N=10 (Son et al. 2010)  Iscador^®^ M Spezial (n=30/ Follow up study n=28: N=30/ Follow up study N=28 (Tröger et al. 2009, 2012);  Helixor^®^ A (n=34, Follow up study n=28): N=34/ Follow up study N=28 (Tröger et al. 2014b, 2016);  Iscador^®^ M (n=1): N=1 (Wode et al. 2009)  In sum: 21 studies   - 4 studies with different mistletoe preparations - 16 studies with one mistletoe preparation - 1 study with mistletoe preparation from the same pharmaceutical company, but not from the same host tree - Iscador^®^: 16 studies - Helixor^®^: 6 studies (in 1 study Helixor^®^ preparation from 3 different host trees, counted only once) - AbnobaViscum^®^: 2 studies - Lektinol^®^: 2 studies - Iscucin^®^: 2 studies - Eurixor^®^: 1 study |
| Pancreatic cancer | Eurixor^®^ (n=16): N=16 (Friess et al. 1996);  Iscador^®^ (n=201): N=201 (Matthes et al. 2010);  Helixor^®^ NS (n=31), Abnobaviscum^®^ (n=30), Iscador^®^ (n=9); Iscucin^®^ (n=2): N=39 (Schad et al. 2014);  AbnobaViscum^®^: AbnobaViscum^®^ Abietis (n=13)/ Aceris (n=2)/ Fraxini (n=31)/ mali (n=36)/ Pini (n=5)/ Quercus (n=24): N=54 (Thronicke et al. 2020a);  Iscador^®^ Qu/ Qu Spezial (n= 110/ Follow up study n=96): N=110/ Follow up study N=96 (Tröger et al. 2013, 2014a);  AbnobaViscum^®^ Fraxini (n=1) and Iscucin^®^ Salicis (n=1): N=1 (Werthmann et al. 2018b);  Iscador^®^ P (n=1)/ Qu (n=1): N=1 (Werthmann et al. 2019a)  In sum: 7 studies   - 2 studies with different mistletoe preparations - 3 studies with one mistletoe preparation - 2 studies with mistletoe preparation from the same pharmaceutical company, but not from the same host tree - Iscador^®^: 4 studies (in 1 study Iscador^®^ preparation from 2 different host trees, counted only once) - Helixor^®^: 1 study - AbnobaViscum^®^: 3 studies (in 1 study AbnobaViscum^®^ preparation from 6 different host trees, counted only once) - Iscucin^®^: 2 studies - Eurixor^®^: 1 study |
| Colorectal cancer | AbnobaViscum^®^ Quercus (n=25): N=25 (Bar-Sela and Haim 2004);  Iscador^®^ Qu (n=181): N=181 (Bock et al. 2014);  Isorel^®^ A (n=29): N=29 (Cazacu et al. 2003);  Iscador^®^ Qu (n=227)/ M (n=172)/ P or combination of Qu/ M/ P (n=30): N=429 (Friedel et al. 2009);  Iscador^®^ M Spezial (n=11): N=11 (Schink et al. 2007);  Iscador^®^ Qu (n=106): N=106 (Zaenker et al. 2012)  In sum: 6 studies   - 5 studies with one mistletoe preparation - 1 study with mistletoe preparations from the same pharmaceutical company, but not from the same host tree - Isador^®^: 4 studies (in 1 study Iscador^®^ preparation from 3 different host trees, counted only once) - AbnobaViscum^®^: 1 study - Isorel^®^: 1 study |
| Malignant melanoma | Iscador^®^ P (n=274)/ M and/or Qu (n=55): N=329 (Augustin et al. 2005);  Iscador^®^ (n=54): N=54 🡪 one randomized study: N=22, one non-randomized: N=32 (Grossarth-Maticek and Ziegler 2007a);  Iscador^®^ M (n=102): N=102 (Kleeberg et al. 2004);  Iscador^®^ P (n=22)/ P cum Hg (n=11)/ M (n=1)/ NS (n=5), Helixor^®^ P (n=10)/ A (n=2)/ M (n=2)/ NS (n=3), AbnobaViscum^®^ (n=1), Change of preparations (n=9): N=66 (Stumpf et al. 2003);  Iscador^®^ P cum Hg (n=1)/ P (n=1)/ M (n=1)/ Qu (n=1)/ M Spezial (n=1), VAE Qu F (n=1): N=1 (Werthmann et al. 2017a)  In sum: 6 studies   - 2 studies with different mistletoe preparations - 3 studies with one mistletoe preparation - 1 study with mistletoe preparation from the same pharmaceutical company but not from the same host tree - Iscador^®^: 6 studies (in 2 studies Iscador^®^ preparation from 3 different host trees each, in 1 study Iscador^®^ preparation from 2 different host trees, counted only once) - Helixor^®^: 1 study (Helixor^®^ preparation from 3 different host trees, counted only once) - AbnobaViscum^®^: 1 study - VAE: 1 study |
| Lung cancer | Iscador^®^ Qu (n=33): N=33 (Bar-Sela et al. 2013);  Helixor^®^ M (n=52): N=52 (Lee et al. 2019);  AbnobaViscum^®^ (n=42), Helixor^®^ (n=31), Iscador^®^ (n=12): N= 50 (Schad et al. 2018b);  AbnobaViscum^®^ (n=12), Helixor^®^ (n=20), Iscador^®^ (n=6): N=38 (Thronicke et al. 2020b)  In sum: 4 studies   - 2 studies with different mistletoe preparations - 2 studies with one mistletoe preparation - Iscador^®^: 3 studies - Helixor^®^: 3 studies - AbnobaViscum^®^: 2 studies |
| Renal cell carcinoma | Eurixor^®^ (n=37): N=37 (Brinkmann and Hertle 2004);  Iscador^®^ Qu cum Cu (n=8)/ M cum Cu (n=6): N=14 (Kjaer 1989);  AbnobaViscum^®^ Fraxini (n=1): N=1 (Reynel et al. 2019);  Helixor^®^ A (n=1): N=1 (Werthmann et al. 2019b)  In sum: 4 studies   - 3 studies with one mistletoe preparation - 1 study with mistletoe preparations from the same pharmaceutical company, but not from the same host tree - Iscador^®^: 1 study (Iscador^®^ preparation from 2 different host trees, counted only once) - Helixor^®^: 1 study - AbnobaViscum^®^: 1 study - Eurixor^®^: 1 study |
| Bladder cancer | misletoe extract (n=30): N=30 (Elsasser-Beile et al. 2005a);  ML (n=23): N=23 (Goebell et al. 2002);  AbnobaViscum^®^ Fraxini (n=36): N=36 (Rose et al. 2015)  In sum: 3 studies   - 3 studies with one mistletoe preparation - AbnobaViscum^®^: 1 study - ML: 1 study - mistletoe extract: 1 study |
| Ovarian cancer | Iscador^®^ (n=178): N=178 🡪 two randomized studies: N=20, N=41, two non-randomized: N=75, N=62 (Grossarth-Maticek and Ziegler 2007c);  mistletoe extract (n=1): N=1 (Hwang et al. 2019);  VAE Pini (n=1)/ Mali (n=1): N=1 (Werthmann et al. 2018c)  In sum: 6 studies   - 5 studies with one mistletoe preparation - 1 study with mistletoe preparations from the same pharmaceutical company, but not from the same host tree - Iscador^®^: 4 studies - VAE/ mistletoe extract: 2 (in 1 study VAE from 2 different host trees, counted only once) |
| Osteosarcoma | Iscador^®^ P (n=9): N=9 (Longhi et al. 2014, 2020)  In sum: 1 study   - 1 study with one mistletoe preparation - Iscador^®^: 1 study |
| Cervical cancer | Iscador^®^ Mali cum Arg (n=81): N= 81 (Fellmer 1968);  Iscador^®^ (n=187): N=187 🡪 one randomized study: N=19, two non-randomized studies: N=102, N=66 (Grossarth-Maticek and Ziegler 2007b);  AbnobaViscum^®^ Fraxini (n=1): N=1 (Reynel et al. 2018)  In sum: 5 studies   - 5 studies with one mistletoe preparation - Iscador^®^: 4 studies - AbnobaViscum^®^: 1 study |
| Glioma | Eurixor^®^ (n=20): N=20 (Lenartz et al. 2000)  In sum: 1 study   - 1 study with one mistletoe preparation - Eurixor^®^: 1 study |
| Gastric cancer | Iscador^®^ (n=67): N=67 (Günczler et al. 1968);  AbnobaViscum^®^ Quercus (n=16, analyzed: 15): N=16, analyzed: 15 (Kim et al. 2012);  VAE (n=1): N=1 (Oh 2020)  In sum: 3 studies   - 3 studies with one mistletoe preparation - AbnobaViscum^®^: 1 study - Iscador^®^: 1 study - VAE: 1 study |
| Liver cancer (HCC) | Viscum-Fraxini-2^®^ (n=120): N=120 (Ebrahim et al. 2010);  Viscum-Fraxini-2^®^ (n=23): N=23 (Mabed et al. 2004)  In sum: 2 studies   - 2 studies with one mistletoe preparation - Viscum-Fraxini-2^®^: 2 studies |
| Head and neck cancer | Eurixor^®^ (n=235/ Follow up study n=200): N=235/Follow-up study N=200 (Steuer-Vogt et al. 2001, 2006)  In sum: 1 study   - 1 study with one mistletoe preparation - Eurixor^®^: 1 study |
| DLBCL | Helixor^®^ P (n=1): N=1 (Gutsch et al. 2018)  In sum: 1 study   - 1 study with one mistletoe preparation - Helixor^®^: 1 study |
| Thymic neuroendocrine tumor (TNET) | AbnobaViscum^®^ Fraxini (n=1): N=1 (Reynel et al. 2020)  In sum: 1 study   - 1 study with one mistletoe preparation - AbnobaViscum^®^: 1 study |
| Rectal carcinoma | AbnobaViscum^®^ Fraxini (n=1): N=1 (Werthmann et al. 2018a)  In sum: 1 study   - 1 study with one mistletoe preparation - AbnobaViscum^®^: 1 study |
| Merkel cell carcinoma | Helixor^®^ A (n=1): N=1 (Werthmann et al. 2018d)  In sum: 1 study   - 1 study with one mistletoe preparation - Helixor^®^: 1 study |
| Adenoid cystic carcinoma | AbnobaViscum^®^ Quercus (n=1)/ Fraxini (n=1), Iscador^®^ Qu cum Ag (n=1): N=1 (Werthmann et al. 2014)  In sum: 1 study   - 1 study with with different mistletoe preparations - AbnobaViscum^®^: 1 study (in 1 study AbnobaViscum^®^ preparation from 2 different host trees, counted only once) - Iscador^®^: 1 study |
| Mesothelioma | AbnobaViscum^®^ Pini (n=1)/ Fraxini (n=1), Helixor^®^ P (n=1), Iscador^®^ cum Hg (n=1): N=1 (Werthmann et al. 2017b)  In sum: 1 study   - 1 study with different mistletoe preparations - Iscador^®^: 1 study - Helixor^®^: 1 study - AbnobaViscum^®^: 1 study (in 1 study AbnobaViscum^®^ preparation from 2 different host trees, counted only once) |
| Corpus uteri cancer | Iscador^®^ (n=254): N=254 🡪 two randomized studies: N=30, N=26; two non-randomized studies: N=103, N=95 (Grossarth-Maticek and Ziegler 2008)  In sum: 4 studies   - 4 studies with one mistletoe preparation - Iscador^®^: 4 studies |
| Hematological and lymphatic cancers | Helixor^®^ A (n=57)/ P (n=69)/ M (n=7)/ NS (n=39), Iscador^®^ P (n=16)/ P cum Hg (n=2)/ Q (n=2)/ NS (n=23), Eurixor^®^ (n=2), AbnobaViscum^®^ Fraxini (n=7)/ NS (n=3), Isorel^®^ A (n=1)/ P (n=2), Plenosol^®^ (n=1): N=223 (Stumpf et al. 2000)  In sum: 1 study   - 1 study with different mistletoe preparations - Iscador^®^: 1 study (Iscador^®^ preparation from 2 different host trees, counted only once) - Helixor^®^: 1 study (Helixor^®^ preparation from 3 different host trees, counted only once) - AbnobaViscum^®^: 1 study - Isorel^®^: 1 study (Isorel^®^ preparation from 2 different host trees, counted only once) - Plenosol^®^: 1 study |
| Histiocytosis | Helixor^®^ A (n=1): N=1 (Seifert et al. 2007)  In sum: 1 study   - 1 study with one mistletoe preparation - Helixor^®^: 1 study |
| Malignant pleural effusion | AbnobaViscum^®^ Fraxini (n=1): N=1 (Cho and Kim 2018)  In sum: 1 study   - 1 study with one mistletoe preparation - AbnobaViscum^®^: 1 study |
| Various cancer types | Iscador^®^ M (n=23): N=23 (Bar-Sela et al. 2006);  Iscador^®^ Qu/ M/ P (n = 13), AbnobaViscum^®^ Quercus/ Abietis (n = 11), Isorel^®^ (n = 1): N=25 (Brandenberger et al. 2012);  AbnobaViscum^®^ Fraxini (n=62), N=62 (Cho et al. 2016);  Viscum-Fraxini-2^®^ (Abnoba) 🡪 AbnobaViscum Fraxini^®^ (n=15): N=15 (El-Kolaly et al. 2016);  Isorel^®^ A (n=40): N=40 (Enesel et al. 2005);  AbnobaViscum^®^ (n=3): N=3 (Eom et al. 2017);  AbnobaViscum^®^: N=43 (Eom et al. 2018);  Viscum-Fraxini-2^®^ (Atos pharma) (n=13) (N=13, Gaafar et al. 2014);  VAE Quercus (Weleda) 🡪 Iscador^®^ (n=4): N=4 (Gardin 2009);  Helixor^®^ P (n=21): N=21 (Huber et al. 2017);  Helixor^®^ A (n=117, analyzed: 114): N=117, analyzed: 114 (Klose et al. 2003);  Iscador^®^ (n=124): N=124 (Majewski and Bentele 1963);  AbnobaViscum^®^, Helixor^®^, Iscador^®^, Iscucin^®^: N=103 (Oei et al. 2019b);  Helixor^®^ A (n=118, analyzed: 115): N=118, analyzed: 115 (Piao et al. 2004);  AbnobaViscum^®^ Fraxini (n=822)/ Mali (n=176)/ Quercus (n=213)/ all other AbnobaViscum^®^ preparations (Abietis, Aceris, Amygdali, Betulae, Crataegi and Pini) (n=150): N= 1361 (Schad et al. 2017);  Helixor^®^ A (n=38)/ M (n=20)/ P (n=6): N=55 (Schad et al. 2018a);  Iscador^®^ P/ M/ Qu/ A/ U, AbnobaViscum^®^ Fraxini, Helixor^®^ P: N=59 (Schläppi et al. 2017);  AbnobaViscum^®^ (n=1315), Iscador^®^ (n=444), Helixor^®^ (n=323), Iscucin^®^ (n=67), Lektinol^®^ (n=12), Isorel^®^ (n=5), Eurixor^®^ (n=1): fraxini, mali, quercus, pini, abietis, aceris, betulae, ulmi, salicis, amygdali. populi, cratagi and tiliae: N=1923 (Steele et al. 2014a);  Helixor^®^ total (n=321): Helixor^®^ NS (n=5)/ A/ (n=106)/ M (n=187)/ P (n=51), Iscador^®^ total (n=40): Iscador^®^ NS (n=4)/ M(n=8)/ M cum Arg (n=1)/ M Special (n=8)/ P (n=2)/ Qu (n=9)/Qu Special (n=9)/ Qu ulmi cum Hg (n=2), AbnobaViscum^®^ total (n=161): AbnobaViscum^®^ NS (n=2)/ Abietis (n=15)/ Aceris (n=2)/ Amygdali (n=2)/ Crategi (n=1)/ Fraxini (n=133)/ Mali (n=2)/ Pini (n=4)/ Quercus (n=7): N=2805 (Steele et al. 2014b);  AbnobaViscum^®^ total (n=75): AbnobaViscum^®^ Fraxini (n=71)/ NS (n=3)/ Aceris (n=1)/ Mali (n=2)/ Quercus (n=4), Helixor total (n=56): Helixor^®^ M (n=54)/ NS (n=2)/ A (n=1)/ P (n=1), Iscucin^®^ Tiliae (n=1): N= 123 (Steele et al. 2015);  AbnobaViscum^®^ Fraxini (n=7)/ Amygdali (n=1), Helixor^®^ P (n=1), Iscador^®^ Qu (n=1): N=9 (Thronicke et al. 2017);  AbnobaViscum^®^ total (n=184): AbnobaViscum^®^ Abietis (n=4)/ Aceris (n=6)/ Craetegi (n=2)/ Fraxini (n=119)/ Mali (n=20)/ Quercus (n=33)/ Pini (n= 6): N=184 (Thronicke et al. 2018);  AbnobaViscum^®^ Fraxini (n=10): N=10 (Zuzak et al. 2018)  In sum: 22 studies   - 7 studies with different mistletoe preparations - 12 studies with one mistletoe preparation - 3 studies with mistletoe preparations from the same pharmaceutical company, but not from the same host tree - Iscador^®^: 9 studies (in 2 studies Iscador^®^ preparation from 3 different host trees, in 1 study Iscador^®^ preparation from 5 different host trees, counted only once) - Helixor^®^: 10 studies (in 3 studies Helixor^®^ preparation from 3 different host trees each) - AbnobaViscum^®^: 14 studies (in 2 studies AbnobaViscum^®^ preparation from 2 different host trees each, in 1 study AbnobaViscum^®^ preparation from 9 different host trees, in 1 study AbnobaViscum^®^ preparation from 8 different host trees, in 1 study AbnobaViscum^®^ preparation from 4 different host trees, in 1 study AbnobaViscum^®^ preparation from 7 different host trees, counted only once) - Viscum-Fraxini^®^: 1 study - Isorel^®^: 3 studies - Lektinol^®^: 1 study - Eurixor^®^: 1 study - Iscucin^®^: 3 studies |

n: Number of patients exposed to a specific mistletoe preparation, N: Total number of VA patients (Intervention group)

The numbers of the references refer to the reference list in the main manuscript.
