# Supplementary material for: Systematic analysis of mistletoe prescriptions in clinical studies
Source: J Cancer Res Clin Oncol. 2022 Dec 9;149(9):5559–71. doi: 10.1007/s00432-022-04511-2 (PMC10356894; doi:10.1007/s00432-022-04511-2)
Supplement: Supplementary file 8 — Supplementary file8 (DOCX 213 KB) [file 432_2022_4511_MOESM8_ESM.docx]

**Systematic analysis of mistletoe prescripitions in clinical studies**

Henrike Staupe^1^, Judith Buentzel^2^, Christian Keinki^1^, Jens Buentzel^3^, Jutta Huebner^1^

^1^ Klinik für Innere Medizin II; Hämatologie und Onkologie, Universitätsklinikum Jena

^2^Klinik für Hämatologie und medizinische Onkologie, Universitätsmedizin Göttingen

^3^Klinik für HNO-Erkrankungen, Südharz-Klinikum Nordhausen

Corresponding author: Henrike Staupe. h.staupe@web.de

Journal: Journal of cancer research and clinical oncology

**Table e7** Type of application

| Type of application |  | Dosage |
| --- | --- | --- |
| Subcutaneous |  | Iscador^®^ P/ M and/or Qu: 2−3 times, for ≥3 months, no data on dose (Augustin et al. 2005);  AbnobaViscum^®^ Quercus: 3 times/ week, in daily increasing doses from 0.15 to 15 mg; first 4 weeks: lower doses (0.15 mg plant extract (level 4) during the first 2 weeks and 1.5 mg during the next 2 weeks) (Bar-Sela and Haim 2004);  Iscador^®^ Qu: 3 times/week, day 1: Serie 0 (0,01–1 mg) given on seven consecutive days as a hypersensitivity test; Day 9: 10 mg, given every other day until tumour progression (Bar-Sela et al. 2013);  Iscador^®^: 2-3 times/ week for ≥ 3 months, mean cumulative mistletoe extract dose: 4,367 mg (Bock et al. 2004a);  Iscador^®^ Qu: total average dosage of 16,0 mg to 20,0 mg per week (Bock et al. 2014);  Iscador^®^ Qu/ M/ P, AbnobaViscum^®^ Quercus/ Abietis, Isorel^®^ 🡪 2 or 3 times/ week, no data on dose (Brandenberger et al. 2012);  Eurixor^®^ 1ml: 2 times/ week (Monday + Thursday) (Brinkmann and Hertle 2004);  Viscum-Fraxini-2^®^: 1 ml of Viscum-Fraxini^®^ in dilution stage-2 (15 mg extract of 20 mg mistletoe herb from ash tree, diluted in di-natrium-monohydrogen phosphate, ascorbic acid and water) which is equivalent to 10 000 ng/mL injection ampoules: two ampules once weekly (Ebrahim et al. 2010);  Isorel^®^ A 60 mg: two weeks before and after sugery, 6 vials per week as follows (1 vial containing 60 mg): first pre-operative week: 1 vial, then 2 vials, then 3 vials every second day, second pre-operative week: same dosage, but in descending order, postoperative treatments: repeated in the same manner (Enesel et al. 2005);  Iscador^®^ Mali cum Arg: 2 or 3 times/ week, in courses of various concentrations (called “Strengths”), every course of injections starts with one of the higher dilutions (i.e. lower concentrations) and gradually changes to the lower Strengths (i.e. higher concentrations), one course consists of 14 (to 21) injections, Start: Strength 5, change to Strength 4 and Strength 3 and sometimes Strength 2 🡪 typical sequence: Strength 5, 5, 4, 4, 3, 3, 3; after break for one week: injections are repeated in the same sequence, treated for 3 years (Fellmer 1968);  Iscador^®^ Qu/ M/ P or combination of Qu/ M/ P: 2-3 times/ week (Friedel et al. 2009);  Eurixor^®^: twice per week, beginning with 0,1 ml, increasing dose to 1ml within 14 days (Friess et al. 1996);  Iscador^®^ Qu 20 mg: seven doses/ injections, 2 times/ week (interval of 3 or 4 days) (Gardin 2009);  ML 1 ml: twice per week (days 2 and 4) for 3 months, followed by a break of 3 months before second cycle (Goebell et al. 2002);  Iscador^®^ QuFrF/ Qu Spezial: twice 0.1 mg and once 2,5 mg each (Gorter et al. 1998);  Iscador^®^: 2-3 times/ week, no data on dose (Grossarth-Maticek and Ziegler 2006a, 2007b, 2007c, 2008);  Iscador^®^: starting immediately postoperatively, in increasing concentrations from 0, 1 to 3%, given in Series (Serie: 14 vials), injections at 2- to 3-day intervals, between the individual series: pauses of initially 2 to 3 weeks, which can gradually be prolonged (Günczler et al. 1968);  Iscador^®^: concentrations used range from 0.01% (strength 4) to 3%; in severe cases even 5% (Günczler and Salzer 1969);  Helixor P^®^: beginning with 1 mg, 3 times/ week, Month 8: 5 mg, followed by increasing dosages: 10 mg, 20 mg, 30 mg, up to 50 mg (Gutsch et al. 2018);  Iscador^®^ Qu Spezial/ P: two times/ week for 12 weeks at intervals of 3 and 4 days in increasing doses according to the recommendations of the manufacturer 🡪 Week 1: 0,01 mg IP, 0,01 mg IQ Week 2: 0,1 mg IP, 0,1 mg IQ Week 3: 1 mg IP, 1 mg IQ Week 4: 1 mg IP, 1 mg IQ Week 5: 10 mg IP, 2,5 mg IQ Week 6: 10 mg IP, 2,5 mg IQ Week 7: 20 mg IP, 5 mg IQ Week 8: 20 mg IP, 5 mg IQ Week 9: 20 mg IP, 5 mg IQ Week 10: 20 mg IP, 5 mg IQ Week 11: 20 mg IP, 5 mg IQ Week 12: 20 mg IP, 5 mg IQ (Huber et al. 2002);  Iscucin^®^ Populi: in increasing doses: strength F, G,and H (each for 4 weeks), 2 times/ week over a period of 12 weeks and Viscum mali e planta tota^®^: in increasing doses: 1:1000 [D3, containing 1 mg mistletoe extract], 1:100 [D2, containing 10 mg mistletoe extract], 1:50 (2%, containing 20 mg mistletoe extract) (each for 4 weeks), 2 times/ week over a period of 12 weeks and Viscum mali e planta tota^®^ (1:1000 [D3], 1:100 [D2] and 2% each for 4 weeks): 2 times/ week over a period of 12 weeks (Huber et al. 2011);  mistletoe extract: in increasing doses: 1 to 20mg/mL for nearly 21 months (Hwang et al. 2019)*;  AbnobaViscum^®^ Quercus: 3 times/ week from postop day 7 to week 24 in increasing doses: 8 x 0,02 mg, followed by 8 x 0,2 mg, 8 x 2 mg and 8 x 20mg, continued with 20mg to the end of the study 🡪 this schedule corresponds to the recommended treatment schedule of the manufacturer (Kim et al. 2012);  Iscador^®^ Qu cum Cu/ M cum Cu: 3 times/ week, in increasing doses: week 1: 0,1 mg/ml, 1 mg/ml, 10 mg/ml, week 2: 1 mg/ml, 10 mg/ml, 20 mg/ml, week 3: 10 mg/ml, 20 mg/ml, 30 mg/ml, week 4: 20 mg/ml, 30 mg/ml ,50 mg/ml (Kjaer 1989);  Iscador^®^ M: beginning at dose level 0, then 0,01 to 1,0 mg/ml every other day for 2 weeks, 3 days no treatment, repeat until 14 doses 20 mg/ml, 7 days no treatment, according to the summary of product characteristics (Kleeberg et al. 2004);  Helixor^®^ A: 3 times /week (day 1, 3, 5), Week 1: 1 mg, 5 mg, 10 mg Week 2: 10 mg, 20 mg, 20 mg Week 3: 30 mg, 30 mg, 50 mg Week 4: 50 mg; 70 mg, 70 mg Week 5: 80 mg, 80 mg, 100 mg Week 6-8: 100 mg; 150 mg; 200 mg (Klose et al. 2003);  Eurixor^®^: 1 ng ML-1/kg body weigt, 2 times/ week for 3 months, from the first day after surgery (Lenartz et al. 2000);  Iscador^®^: in increasing doses, given in Series of 14 iniections of different strengths, amount of substance (fresh plant) per series averages 70-80 mg (Leroi 1977);  Iscador^®^ M Spezial 5 mg: week 1 +2: 1 ampoule IM Serie 0 (0,01–1 mg) daily, followed by: 1/2 ampoule (2,5 mg), 2 times/ week (Loewe-Mesch et al. 2008);  Iscador^®^ P: 3 times/ week for 12 months, beginning with two boxes of Series 0 (each box contains 2 vials of 0.01 mg, 2 vials of 0.1 mg, and 3 vials of 1 mg), followed by two boxes of series I (2 vials of 0.1 mg, 2 vials of 1 mg, and 3 vials of 10 mg vials), subsequent treatment with series II (1, 10, and 20 mg) continuously until the 12th month (Longhi et al. 2014, 2020);  Viscum-Fraxini-2^®^: 2 ampoules once weekly, one milliliter of Viscum-Fraxini in dilution stage–2 (15 mg extract of 20 mg mistletoe herb from ash tree, diluted in di-natrium-mono-hydrogen phosphate, ascorbic acid and water) which is equivalent to 10 000 ng/ml injection ampoules (Mabed et al. 2004);  Iscador^®^: at 2 day intervals, no data on dose (Majewski and Bentele 1963);  Iscador^®^: 2-3 times/ week, according to the producer´s recommendations (Matthes et al. 2010);  AbnobaViscum^®^, Iscador^®^, Helixor^®^, Iscucin^®^: no data on dose (Oei et al. 2018)*;  AbnobaViscum^®^, Helixor^®^, Iscador^®^, Iscucin^®^: application according to summary of product characteristics (Oei et al. 2019a)*  AbnobaViscum^®^. Iscador^®^, Helixor^®^, Iscucin^®^: 2 to 3 times/ week doses in increasing doses, Subgroup analysis: over a period of at least 6 months, high s.c. VA-applications: AbnobaViscum^®^ >0.2 mg/mL , Iscador^®^ ≥1 mg/mL or Helixor^®^ ≥10 mg/mL, Iscucin^®^: no data on dose (Oei et al. 2019b)*;  VAE: 3 times/ week, no data on dose (Oh 2020);  Helixor^®^ A: in increasing doses: 3 x 1 mg, 3 x 5 mg, 3 x 10mg, 3 x 20 mg, 3 x 30 mg, remaining doses: 50 mg and Iscador^®^ M Spezial: in increasing doses: 2 x 0.01 mg, 2 x 0.1 mg , 11 x 1 mg , 8 x 2 mg, remaining doses: 5 mg 🡪 3 times/ week (e.g., Monday, Wednesday, and Friday) (Pelzer et al. 2018);  Helixor^®^ A: 3 times/ week, increasing doses: starting with 1 mg to 200 mg (Piao et al. 2004);  AbnobaViscum^®^ Fraxini 🡪 3 times/ week: Month 1: 0,2 mg s.c. (7 vials) Month 2: 0,2 mg s.c. (7 vials), 2 mg s.c. (3 vials) Month 3: 2 mg s.c. (10 vials), 20 mg s.c. (1 vial) Month 4: 20 mg s.c. (9 vials) Month 5: 20 mg s.c. (8 vials) Month 6: 20 mg s.c. (8 vials) Month 7: 20 mg s.c. (9 vials) Month 8: 20 mg s.c. (12 vials) Month 9: 20 mg s.c. (8 vials) Month 10: 20 mg s.c. (12 vials) Month 11: 20 mg s.c. (9 vials) Month 12: 20 mg s.c. (6 vials) Month 13-36: 20 mg (8 vials) (Reynel et al. 2018)*;  AbnobaViscum^®^ Fraxini: up to 3 times/week in increasing doses, Month 3 (0,2 mg → 20 mg, 2 times/ week): week 1 (0,2 mg), week 2 (2 mg), week 3 and 4 (20 mg); Month 7 (0.2 mg → 20 mg, 1 time/ week): week 1 (0,2 mg), week 2 (2 mg)., week 3 and 4 (20 mg); Month 8 (20 mg, 2 times/ week); Month 9-18 (20 mg, 3 times/ week); Month 20 (2 mg → 20 mg, 3 times/ week): week 1 (0,2 mg), week 2 (2 mg), week 3 and 4 (20 mg); Month 21-30 (20 mg, 3 times/ week) (Reynel et al. 2019)*;  AbnobaViscum^®^ Fraxini: 3 times/ week for 85 months in increasing doses: beginning with 0,2 mg, week 2: 2mg for 40 months, month 41: 20mg for the folllowing 44 months (Reynel et al. 2020);  Helixor^®^ NS, AbnobaViscum^®^, Iscador^®^, Iscucin^®^: no data on dose (Schad et al. 2014)*;  AbnobaViscum^®^ Fraxini/ Mali/ Quercus/ all other AbnobaViscum^®^ preparations (Abietis, Aceris, Amygdali, Betulae, Crataegi and Pini): low dose group: 0.02 mg, high dose group: 0.1 mg to 40 mg (Schad et al. 2017);  AbnobaViscum^®^, Helixor^®^, Iscador^®^: application according to summary of product characteristics (Schad et al. 2018b)*;  Eurixor^®^ 1ml: twice per week, therapy duration: 270 days (Schumacher et al. 2003);  Helixor^®^ A: 3 times/ week, week 1: 1 mg; week 2: 2,5 mg, week 3: 5 mg; week 4: 1 mg (Seifert et al. 2007);  Lektinol^®^ 0,5ml (containing 10, 30 or 70 ng ML/ml): 2 times/ week for 15 consecutive weeks (Semiglasov et al. 2004);  Lektinol^®^ 0.5 ml (containing 30 ng ML/ml): 2 times/ week (on days 1 and 4 of each week) for 16 to 24 consecutive days (Semiglazov et al. 2006);  Iscador^®^ M: 3 times/ week, no data on dose (Shaw et al. 2004);  Helixor^®^: 3 times/ week, for seven weeks, in increasing doses: 1 mg up to 100 mg (Series): week 1: 3 x 1mg, week 2: 3 x 5mg, week 3: 3 x 10 mg, week 4+5: 3 x 20mg, week 6: 3 x 50mg, week 7: 3 x 100 mg (Son et al. 2010);  AbnobaViscum^®^, Iscador^®^, Helixor^®^, Iscucin^®^, Lektinol^®^, Isorel^®^, Eurixor^®^ (fraxini, mali, quercus, pini, abietis, aceris, betulae, ulmi, salicis, amygdali. populi, cratagi, and tiliae): 3 times/ week, according to the summary of product characteristics for each of the products, beginning with a very low or low dose of mistletoe extract, increasing to a moderate or high dose over time: AbnobaViscum^®^: very low (≤0.02 mg/Ml), low (>0.02–0.2 mg/mL), moderate (>0.2–2.0 mg/mL), high (>2.0 mg/mL) Helixor^®^: very low (<1 mg/mL), low (1–<10 mg/mL), moderate (10–<30 mg/mL), high (≥30 mg/mL) Iscador^®^: very low (≤0.01 mg/mL), low (>0.01–<1 mg/mL), moderate (1–<10 mg/mL), high (≥10 mg/mL) Iscucin^®^: very low (Strengths A and B), low (Strengths C and D), moderate (Strengths E and F), high (Strengths G and H), Isorel^®^: very low (Strength 1), low (Strengths 6 and 12), moderate (Strengths 24 and 32), high (Strength 60), Lektinol^® a^, Eurixor^® b^ (Steele et al. 2014a);  Eurixor^®^: 1 ng ML-1/kg body weight, twice per week for 60 weeks, in intervals of 12 weeks, 4 weeks pause) as recommended by the manufacturer (Steuer-Vogt et al. 2001, 2006);  Helixor^®^ A/ P/ M/ NS, Iscador^®^ P/ P cum Hg/ Q/ NS, Eurixor^®^, AbnobaViscum^®^ Fraxini/ NS, Isorel^®^ A/ P, Plenosol^®^: average duration of therapy: 2.68 years (median 1.68 years), no data on dose (Stumpf et al. 2000);  AbnobaViscum^®^ Fraxini 0,2 mg, AbnobaVIscum^®^ Amygdali 0,2 mg, Iscador^®^ Qu 5 mg: according to the summary of product characteristics (Thronicke et al. 2017)*;  AbnobaViscum^®^: AbnobaViscum^®^ Abietis/ Aceris/ Craetegi/ Fraxini/ Mali/ Quercus/ Pini: according to the summary of product characeteristics (Thronicke et al. 2018)*;  AbnobaViscum^®^: AbnobaViscum^®^ Abietis/ Aceris/ Fraxini/ Mali/ Pini/ Quercus: according to the summary of product characteristics (Thronicke et al. 2020a)*;  AbnobaViscum^®^, Helixor^®^, Iscador^®^: no data on dose (Thronicke et al. 2020b)*  Iscador^®^ M Spezial: 3 times/ week (i.e. Monday, Wednesday, Friday) within 18 weeks, dose escalation schedule: 2 × 0,01 mg, 2 × 0,1 mg, 11 × 1 mg, 8 × 2 mg, remaining doses of 5 mg, according to the guidelines of the manufacturer (Tröger et al. 2009, 2012);  Iscador^®^ Qu/ Qu Spezial: 3 times/ week, increasing doses from 0.01 mg up to 10 mg: 2 x 0,01 mg, 2 x 0,1 mg, 5 x 1 mg, 5 x 2 mg and 8 x 5 mg, remaining doses of 10 mg (Tröger et al. 2013, 2014a);  Helixor^®^ A: 3 times/ week, dose escalation schedule: 3 × 1 mg, 3 × 5 mg, 3 × 10 mg, 3 × 20 mg, 3 × 30 mg, remaining doses of 50 mg (Tröger et al. 2014b, 2016);  Iscador^®^ Qu cum Ag: no data on dose (Werthmann et al. 2014)*;  Iscador^®^ P cum Hg: Series I (vials of 0.1–10mg), 3 times/ week and Iscador^®^P 60mg: 3 times/ week (Werthmann et al. 2017a)*;  AbnobaViscum^®^ Pini D10 (every other day)/ AbnobaViscum Fraxini 20 mg (2 times/ week), Helixor P 1-10 mg (every other day/ 2 times/ week)/ 10-30 mg (2 times/week)/ 100 mg (1 or 2 times/ week)/ 200 mg ( 2 times/ week), Iscador cum Hg 20 mg (2 times/ week) (Werthmann et al. 2017b)*;  AbnobaViscum^®^ Fraxini 40 mg, Iscucin^®^ Salicis Strength A and H (Werthmann et al. 2018b)*;  VAE Pini/ Mali: 3 times/ week, with treatment breaks: 1 month out of 3 🡪 Month 0-6: VAE Pini 1 mg; Month 9-13: VAE Pini 1 mg - 20 mg; Month 13 - year 3: VAE Mali 20 mg; Year 3 - present (y 20): VAE Mali 20 mg (Werthmann et al. 2018c);  Helixor^®^ A: 7 injections in increasing dosages (Series I, II or IV) followed by 7 injections in decreasing dosages (Series I, II or IV, each backwards), with a subsequent treatment break of 2 weeks; Serie I:7 vials of lower dosage (3×1mg/mL, 3×5mg/mL, 1×10mg/mL); Serie II: 7 vials of medium dosage (2×10mg/mL, 2×20mg/mL, 3×30mg/mL); Serie IV: 7 vials of higher dosage (2×20mg/mL, 2×30mg/mL, 3×50mg/ mL) Month 1-4: Series I (1 mg/ 5 mg/ 10 mg), 3 times/ week Month 5-6: Series II (10 mg/ 20 mg/ 30 mg), 3 times/ week Month 6-24: Series IV (20 mg/ 30 mg/ 50 mg), 3 times/ week Month 25-74: Series IV (20 mg/ 30 mg/ 50 mg), 2 times/ week. Month 74-86: treatment break Month 87-156: Series IV (20 mg/ 30 mg/ 50 mg), 2 times/ week Month 157-160: treatment break Month 161-192: Series IV (20 mg/ 30 mg/ 50 mg), 2 times/ week Month 193-228: Series IV (20 mg/ 30 mg/ 50 mg), 2 cycles per year (Werthmann et al. 2018d);  Helixor^®^ A 100 mg, 2 times/ week and 1-10 mg, once weekly (Werthmann et al. 2019b)*;  Iscador^®^ M: 3 times/ week (e.g. Monday, Wednesday, Friday) according to mainly general recommendations, i.e. Series 0, Series 1, or Series 2 injections (Series 2 not used in this case), Series 0: 7 injections at a lower dosage (2 × 0.01 mg/ml, 2 × 0.1 mg/ml, 3 × 1 mg/ml); Series 1: 7 injections at higher dosage (2 × 0.1 mg/ml, 2 × 1 mg/ml, 3 × 10 mg/ml); Start: repeated injections of Series 0 for five weeks, followed: by a one-week pause, After this break: depending on the patient's response, either Series 0 is repeated, or the next series commences (Wode et al. 2009);  Iscador^®^ Qu: 2-3 times/ week, median dose: 4,221 mg, (range 696-10,523 mg), estimated mean weekly dose: 16,2 mg, median therapy duration: 54 (11-141) months (Zaenker et al. 2012);  AbnobaViscum^®^ Fraxini: in increasing dosages: 0,02 mg, 0,2 mg, 2 mg, 4 mg, 10 mg, 15 mg etc. (0.02 mg–40 mg) (Zuzak et al. 2018)*  79 publications, 81 studies  🡪 only s.c. application: 61, s.c. application and at least one another application form: 18 |
| Intravenous |  | Isorel^®^ A: 5 mg/kg in saline infusion 500 mL, 3 times/ week (Monday, Wednesday, and Friday) during entire postoperative period (Cazacu et al. 2003);  Helixor^®^ P: one ampoule of 2 ml solution for injection contains 100 mg mistletoe extract, diluted with 250 ml physiologic saline solution 🡪 classical phase I 3 + 3 dose escalation schedule, dose groups: 200 mg, 400 mg, 700 mg, 1200 mg and 2000 mg, once weekly for 3 weeks (Huber et al. 2017);  AbnobaViscum^®^, Iscador^®^, Helixor^®^, Iscucin^®^: no data on dose (Oei et al. 2018)*;  AbnobaViscum^®^, Helixor^®^, Iscador^®^, Iscucin^®^: no data on dose (Oei et al. 2019a)*;  AbnobaViscum^®^, Helixor^®^, Iscador^®^, Iscucin^®^: administered doses and periods varied markedly, high i.v. applications > 300 mg (Oei et al. 2019b)*;  AbnobaViscum^®^ Fraxini: up to 3 times/ week in increasing doses 🡪 Month 1 (20 mg → 160 mg, 3 times/ week): week 1 (20 mg, 40 mg, 60 mg), week 2 (80 mg, 100 mg, 120 mg), week 3 (140 mg, 160 mg, 160 mg), week 4 (160 mg); Month 2: (160 mg, 3 times/ week); Month 3: week 1 (160 mg, once); Month 4-6 (160 mg, 3 times/ week); Month 7: week 1 (160 mg, 2 times/ week); Month 8 (160 mg, once); Month 19 (20 mg → 160 mg, 3 times/ week): week 1 (20 mg, 40 mg, 60 mg), week 2 (80 mg, 100 mg, 120 mg), week 3 (140 mg, 160 mg, 160 mg), week 4 (160 mg) (Reynel et al. 2019)*;  Helixor^®^ NS, AbnobaViscum^®^, Iscador^®^, Iscucin^®^: no data on dose (Schad et al. 2014)*;  Helixor^®^ A/ M/ P: no data on dose (Schad et al. 2018a);  AbnobaViscum^®^, Helixor^®^, Iscador^®^: no data on dose (Schad et al. 2018b)*;  Iscador^®^ M Spezial 5 mg (1ml ampoule) diluted in 250 ml sterile isotonic sodium chloride solution (Schink et al. 2007);  Iscador^®^ P (mean+SD: 39 mg ± 27 mg, median/ range: 30 mg /10-140), Iscador M (mean+SD: 25 mg ± 24 mg, median/ range 20 mg/3-140 mg), Iscador Q (mean+SD: 27 mg ± 22 mg, median/ range: 20 mg/ 2-120 mg), Iscador A (mean+SD: 38 mg ± 19 mg, median/ range: 36 mg/ 14-80 mg), Iscador U (mean+SD: 28 mg ± 11 mg, median/ range: 36 mg / 12-40), AbnobaViscum^®^ Fraxini (mean+SD: 141 mg ± 35 mg, median/ range: 160 mg/ 40-200 mg), Helixor^®^ P (mean+SD: 712 mg ± 335 mg, median/range: 900 mg/ 50-1000 mg) (Schläppi et al. 2017);  Helixor^®^: Helixor^®^ NS/ A/ M/ P: 1 mg to 3000 mg (median dose: 200 mg), Iscador^®^: Iscador^®^ NS/ M/ M cum Arg/ M Special/ P/ Qu/ Qu Special/ Qu ulmi cum Hg: 0.1 mg to 100 mg (median dose: 10 mg), AbnobaViscum^®^: Abnobaviscum^®^ NS/ Abietis/ Aceris/ Amygdali/ Crategi/ Fraxini/ Mali/ Pini/ Quercus: 0.02 mg to 400 mg (median dose: 80 mg) (Steele et al. 2014b);  AbnobaViscum^®^ Fraxini 20 mg, 40 mg and 60 mg Helixor^®^ P 200 mg (Thronicke et al. 2017)*;  AbnobaViscum^®^: AbnobaViscum^®^ Abietis/ Aceris/ Craetegi/ Fraxini/ Mali/ Quercus/ Pini: no data on dose (Thronicke et al. 2018)*;  AbnobaViscum^®^: AbnobaViscum^®^ Abietis/ Aceris/ Fraxini/ Mali/ Pini/ Quercus: no data on dose (Thronicke et al. 2020a)*;  AbnobaViscum^®^, Helixor^®^, Iscador^®^: no data on dose (Thronicke et al. 2020b)*;  Iscador^®^ P/ M/ Qu/ M Spezial, VAE Qu F: Month 1: Iscador^®^ M 15 mg/ 39 mg/ 45 mg/ 60 mg/ 75 mg. Month 2: Iscador^®^ M 82.5 mg/ 90 mg. Month 3: Iscador^®^ M 105 mg Month 4: 2 times Iscador^®^ M 120 mg and VA QuF 200 mg, Iscador^®^ M 135 mg Month 5: Iscador^®^ M 165 mg and VA QuF 100 mg, Iscador^®^ Qu 600 mg and VA QuF 200 mg Month 6: Iscador^®^ Qu 800 mg and VA QuF 200 mg Month 7: Iscador^®^ Qu 1000 mg and VA QuF 200 mg, Iscador^®^ Qu 1300 mg and VA QuF 200 mg Month 8: 2 times Iscador^®^ Qu 2000 mg and VA QuF 200 mg Month 9: 2 times Iscador^®^ Qu 2000 mg and VA QuF 200 mg. Month 10: Iscador^®^ Qu 1400 mg and VA QuF 200 mg, Iscador^®^ Qu 1400 mg Month 11: Iscador^®^ Qu 1200 mg and VA QuF 100 mg Month 12: Iscador^®^ Qu 1400 mg and VA QuF 100 mg, Iscador^®^ Qu 600 mg and VA QuF 120 mg Month 13: Iscador^®^ M Spezial 240 mg and VA QuF 120 mg Month 14: Iscador^®^ M Spezial 200mg and VA QuF 120 mg and Iscador^®^ P 200 mg Month 18: Iscador^®^ M Spezial 200mg and VA QuF 150 mg and Iscador P 200 mg Month 19: VA QuF 150 mg and Iscador^®^ P 300 mg Month 20: VA QuF 300 mg and Iscador^®^ P 280 mg Month 22: Iscador^®^ Q 400 mg and VA QuF 200 mg and Iscador^®^ P 160 mg, Iscador Q 500 mg and VA QuF 200 mg und Iscador P 200 mg Month 23: Iscador^®^ Q 500 mg and VA QuF 200 mg and Iscador^®^ P 200 mg Month 24: Iscador^®^ Q 600 mg and VA QuF 200 mg and Iscador^®^ P 200 mg (Werthmann et al. 2017a)*;  Helixor^®^ P 100 mg, 1 time/ week (Werthmann et al. 2017b)*;  Helixor^®^ A 200 mg (Werthmann et al. 2019b)*;  AbnobaViscum^®^ Fraxini: beginning with 20 mg/m² body surface area (BSA), dose was increased every day in the following steps: 20, 60, 100, 140 mg/ m² BSA, etc., mean maximum dose at end of therapy: 900 mg/ m² BSA (60–3200 mg/m² BSA), which correlates to an absolute amount of 860 mg (144 mg–2600 mg) of VAE (Zuzak et al. 2018)*  20 publications, 20 studies  🡪 only i.v. application: 6, i.v. application and at least one another application form: 14 |
| Intratumoral/ Intralesional |  | AbnobaViscum^®^, Helixor^®^, Iscador^®^: 30 to 200 mg (Oei et al. 2019b)*;  AbnobaViscum^®^ Fraxini 20 mg: 1 vial (week 1), 1 vial (week 2), 2 vials (week 14), 3 vials (week 15), 4 vials (week 16) (Reynel et al. 2018)*;  Helixor^®^ NS, AbnobaViscum^®^: in each session, the tumor was punctured 1 to 3 times: induction phase of 3 applications, with increasing dosages at the start of the therapy, beginning with AbnobaViscum^®^ 20 mg, Helixor^®^ 50 to 100 mg, every 2 to 3 days, mistletoe dosage was increased: AbnobaViscum^®^ 20-40 mg or Helixor^®^ 100-200 mg, up to a maximum dosage of about AbnobaViscum^®^ 160 mg or Helixor^®^ 1400 mg, Iscador^®^ and Iscucin^®^: no data on dose (Schad et al. 2014)*  AbnobaViscum^®^: no data on dose (Schad et al. 2018b)*;  AbnobaViscum^®^: AbnobaViscum^®^ Fraxini/ NS/ Aceris/ Mali/ Quercus: 0,02 to 250 mg (median dose: 60 mg), Helixor^®^: Helixor^®^ M/ NS/ A/ P: 50 to 1400 mg (median dose: 500 mg) , Iscucin^®^ Tiliae: 2, 5, and 6 ampoules of Strength H 🡪 increasing doses (Steele et al. 2015);  AbnobaViscum^®^: AbnobaViscum^®^ Abietis/ Aceris/ Craetegi/ Fraxini/ Mali/ Quercus/ Pini: no data on dose (Thronicke et al. 2018)*;  AbnobaViscum^®^: AbnobaVisum^®^ Abietis/ Aceris/ Fraxini/ Mali/ Pini/ Quercus: no data on dose (Thronicke et al. 2020a)*;  AbnobaViscum^®^ Quercus 2 mg and 20 mg, AbnobaViscum^®^ Fraxini 20 mg: Month 1: once AbnobaViscum^®^ Quercus 2 mg, 3 times AbnobaViscum^®^ Quercus 20 mg Month 2: 4 times AbnobaViscum^®^ Quercus 20 mg Month 3: 4 times AbnobaViscum^®^ Quercus 20 mg Month 4: 4 times AbnobaViscum^®^ Fraxini 20 mg Month 5: 7 times AbnobaViscum^®^ Fraxini 20 mg Month 6: 4 times AbnobaViscum^®^ Fraxini 20 mg Month 7: 8 times AbnobaViscum^®^ Fraxini 20 mg Month 8: once AbnobaViscum^®^ Quercus 20 mg, 2 times AbnobaViscum^®^ Fraxini 20 mg Month 9: 2 times AbnobaViscum^®^ Quercus 20 mg Month 10: 4 times AbnobaViscum^®^ Quercus 20 mg (Werthmann et al. 2014)*;  VAE Qu F 40 mg, 80 mg, 100 mg, 150 mg and 200 mg (Werthmann et al. 2017a)*;  AbnobaViscum^®^ Fraxini 40 -100 mg (Werthmann et al. 2018a);  AbnobaViscum^®^ Fraxini 80 mg (Werthmann et al. 2018b)*;  AbnobaViscum^®^ Fraxini: no data on dose (Zuzak et al. 2018)*  12 publications, 12 studies  🡪 only i.t. application: 2, i.t. application and at least one another application form: 10 |
| Others: | Intrapleural | AbnobaViscum^®^ Fraxini: 5 ampoules of AbnobaViscum^®^ Fraxini 20mg mixed with 0.9% normal saline (Cho et al. 2016);  AbnobaViscum^®^ Fraxini: two ampoules of AbnobaViscum^®^ Fraxini 100 mg mixed with 200 ml normal saline at day 1, 3 and 8 (Cho and Kim 2018);  AbnobaViscum^®^ Fraxini: 5 ampoules of Viscum-Fraxini-2^®^ (each ampoule containing 20 mg mistletoe extract) mixed with 100 ml of normal saline (El-Kolaly et al. 2016);  AbnobaViscum^®^: no data on dose (Eom et al. 2017)  AbnobaViscum^®^ 60 mg (Eom et al. 2018);  Viscum-Fraxini-2^®^: 5 ampoules of Viscum Fraxini-2^®^ diluted in 10 cc glucose 5% solution, repeated every week until complete dryness of the pleural fluid (maximum therapy duration: 8 weeks) (Gaafar et al. 2014);  Helixor^®^ M 100 mg: if the procedure was not effective (drainage volume > 50 mL/day), the procedure was repeated up to five times every other day with a 100 mg increase in the dose of Helixor^®^ M for each subsequent procedure (Lee et al. 2019);  7 publications, 7 studies  🡪 only intrapleural application: 7 |
|  | Intravesikal | misletoe extract: 6 instillations of 50 ml mistletoe extract (containing ML concentrations between 10 and 5,000 ng/ml) at weekly intervals, three patients per group received a dose, which was then doubled in the next group (Elsasser-Beile et al. 2005a);  AbnobaViscum^®^ Fraxini: 45 to 675 mg, weekly instillations for 6 weeks, classical phase I 3 + 3 dose escalation schedule (Rose et al. 2015)  2 publications, 2 studies  🡪 only intravesical application: 2 |
|  | Intraperioneal | Iscador^®^ M 10 mg diluted in 10-15 ml of normal saline (Bar-Sela et al. 2006);  mistletoe extract: 100-500 mg/ml, 3 times/ week for 3 months (Hwang et al. 2019)*  2 publications, 2 studies  🡪 only intraperitoneal application: 2 |
|  | intrathecal | AbnobaViscum^®^ Fraxini: in increasing doses: 0,02 mg, 0,1 mg, 0,2 mg, 0,4 mg, 0,8 mg, 1 mg, 2 mg to 20 mg (Zuzak et al. 2018)*  1 publication, 1 study  🡪 intrathecal application and at least one another application form: 8 |
|  | Oral | AbnobaViscum^®^, Helixor^®^, Iscador^®^, Iscucin^®^: no data on dose (Oei et al. 2019a)*  1 publication, 1 study  🡪 oral application and at least one another application form: 8 |
| No specification | | Helixor^®^ M/ A/ P: no data on dose (Beuth et al. 2008);  Iscador^®^: no data on dose (Grossarth-Maticek and Ziegler 2006b);  Iscador^®^: no data on dose (Grossarth-Maticek and Ziegler 2007a);  Iscador^®^ P/ P cum Hg/ M/ NS, Helixor^®^ P/ A/ M/ NS, AbnobaViscum^®^: no data on dose (Stumpf et al. 2003);  Iscador^®^ P 10-20 mg, Iscador Qu^®^ 10-20 mg and 40 mg: 3 times/ week (Werthmann et al. 2019a)  5 publications, 9 studies |

*: Studies in which mistletoe preparations were applied by different ways

^a^: 2 only available ampoule size: Lektinol^®^ 0,5 ml

^b^: only available ampoule size: Eurixor^®^ 1 ml

The numbers of the references refer to the reference list in the main manuscript.
