# Supplementary material for: Systematic analysis of mistletoe prescriptions in clinical studies
Source: J Cancer Res Clin Oncol. 2022 Dec 9;149(9):5559–71. doi: 10.1007/s00432-022-04511-2 (PMC10356894; doi:10.1007/s00432-022-04511-2)
Supplement: Supplementary file 9 — Supplementary file9 (DOCX 332 KB) [file 432_2022_4511_MOESM9_ESM.docx]

**Systematic analysis of mistletoe prescripitions in clinical studies**

Henrike Staupe^1^, Judith Buentzel^2^, Christian Keinki^1^, Jens Buentzel^3^, Jutta Huebner^1^

^1^ Klinik für Innere Medizin II; Hämatologie und Onkologie, Universitätsklinikum Jena

^2^Klinik für Hämatologie und medizinische Onkologie, Universitätsmedizin Göttingen

^3^Klinik für HNO-Erkrankungen, Südharz-Klinikum Nordhausen

Corresponding author: Henrike Staupe. h.staupe@web.de

Journal: Journal of cancer research and clinical oncology

**Table e8** Studies categorized by endpoints

| 1 | VAE related toxicity/ adverse events/ side effects/ adverse drug reactions | Augustin et al. (2005);  Bar-Sela and Haim (2004);  Bar-Sela et al. (2013);  Beuth et al. (2008);  Bock et al. (2004a);  Brandenberger et al. (2012);  Brinkmann and Hertle (2004);  Ebrahim et al. (2010);  Elsasser-Beile et al. (2005a);  Friedel et al. (2009);  Friess et al. (1996);  Gardin (2009);  Gorter et al. (1998);  Huber et al. (2002, 2011, 2017);  Kjaer (1989);  Kleeberg et al. (2004);  Klose et al. (2003);  Loewe-Mesch et al. (2008);  Mabed et al. (2004);  Matthes et al. (2010);  Oei et al. (2019b);  Pelzer et al. (2018);  Piao et al. (2004);  Reynel et al. (2018, 2019, 2020);  Rose et al. (2015);  Schad et al. (2014, 2017, 2018a);  Schink et al. (2007);  Schläppi et al. (2017);  Schumacher et al. (2003);  Seifert et al. (2007);  Semiglasov et al. (2004),  Semiglazov et al. (2006);  Shaw et al. (2004);  Steele et al. (2014a, b, 2015);  [Steuer-Vogt et al. (2001)](#_ENREF_80);  Tröger et al. (2009, 2013, 2014b);  Thronicke et al. (2017, 2018);  Werthmann et al. (2014, 2017a, b, 2018a, 2018b, d, 2019b);  Zuzak et al. (2018)  🡪 56 publications, 54 studies |
| --- | --- | --- |
| 2 | Quality of life, Psychosomatic self- regulation, Sense of coherence (Inner coherence and resilience + thermo coherence) | General quality of life  European Organization for Research and Treatment of Cancer Core Quality of Life Questionnaire (EORTC QLQ-C30) (Aaronson et al. 1993): 13 studies  Brandenberger et al. (2012); Longhi et al. (2014); Pelzer et al. (2018); Reynel et al. (2018, 2020); Semiglasov et al. (2004); Steuer-Vogt et al. (2001, 2006); Tröger et al. (2009, 2014a, b)  additional lung cancer module (QLQ-LC13); 1 study (Bar-Sela et al. 2013); additional gastric cancer module (QLQ-STO22): 1 study (Kim et al. 2012); additional systematic side effects (BR23): 1 study (Loewe-Mesch et al. 2008)  Functional Assessment of Cancer Therapy-General (FACT-G) (Cella et al. 1993): 1 study  Semiglazov et al. (2006)  Global Life Quality (GLQ-8) (Coates, Glasziou and McNeil 1990) and Spitzer Quality of Life Uniscale (Spitzer et al. 1981): 2 studies  Semiglasov et al. (2004); Semiglazov et al. (2006)  Functional Living Index-Cancer (FLIC) (Schipper et al. 1984): 1 study  Klose et al. (2003); Piao et al. (2004)  Karnofsky Performance Index (KPI) (Karnofsky DA 1949): 4 studies  Enesel et al. (2005); Friess et al. (1996); Klose et al. (2003); Piao et al. (2004); Semiglazov et al. (2006)  Traditional Chinese Medicine Index (TCM): 1 study  Klose et al. (2003); Piao et al. (2004)  SELT-M (Leiberich et al. 1993): 1 study  Brandenberger et al. (2012)  HLQ Version 2.5 (Kümmell HC 1996): 1 study  Brandenberger et al. (2012)  Quality of life questionaire (von Zerssen 1976): 1 study  Friess et al. (1996)  Visual analogue scale (VAS): 1 study  Kjaer (1989)  Interviews: 2 studies  Brandenberger et al. (2012); Reynel et al. (2020)  Psychosomatic self-regulation:  Psychosomatic self-regulation questionnaire (Grossarth-Maticek, Eysenck and Boyle 1995): 19 studies  Grossarth-Maticek and Ziegler (2006a, b, 2007a, b, c, 2008)  Analogic Scale of Anxiety (psychsomatic score): 1 study  Enesel et al. (2005)  Inner and thermo coherence  Internal coherence scale (ICS) (Kröz et al. 2009): 2 studies  Oei et al. (2018, 2019a)  🡪 28 publications, 38 studies |
| 3 | Tumor response, Tumor remission | Bar-Sela and Haim (2004);  Brinkmann and Hertle (2004);  Ebrahim et al. (2010);  Friess et al. (1996);  Gutsch et al. (2018);  Hwang et al. (2019);  Kjaer (1989);  Klose et al. (2003);  Mabed et al. (2004);  Reynel et al. (2018);  Rose et al. (2015);  Schad et al. (2018b);  Seifert et al. (2007);  Schläppi et al. (2017);  Thronicke et al. (2017);  Werthmann et al. (2014, 2017a, b, 2018a, b, c, 2019a, b);  Zuzak et al. (2018);  🡪 24 publications, 24 studies |
| 4 | Overall Survival/ Tumor-related survival | Augustin et al. (2005);  Bar-Sela and Haim (2004);  Bar-Sela et al. (2013);  Bock et al. (2004a);  Brinkmann and Hertle (2004);  Cazacu et al. (2003);  Ebrahim et al. (2010)  Fellmer (1968);  Friess et al. (1996);  Grossarth-Maticek and Ziegler (2006a, b, 2007a, b, c, 2008);  Günczler et al. (1968);  Günczler and Salzer (1969);  Kjaer (1989);  Kleeberg et al. (2004);  Lenartz et al. (2000);  Leroi (1977);  Longhi et al. (2020);  Mabed et al. (2004);  Majewski and Bentele (1963);  Matthes et al. (2010);  Oh (2020);  Reynel et al. (2020);  Schad et al. (2014, 2018b);  Stumpf et al. (2000, 2003);  Thronicke et al. (2017, 2020a, b);  Tröger et al. (2013);  Werthmann et al. (2017b, 2018a, b, 2019a);  Zuzak et al. (2018)  🡪 40 publications, 53 studies |
| 5 | Disease-free-survival ,  Postrelapse-disease-free-survival  Relapses und metastases,  Recurrence rate,  Tumor progression/ Time-to-tumor-progression,  Progression-free-survival | Augustin et al. (2005);  Bar-Sela and Haim (2004);  Bar-Sela et al. (2013);  Ebrahim et al. (2010);  Elsasser-Beile et al. (2005a);  Friedel et al. (2009);  Goebell et al. (2002),  Grossarth-Maticek and Ziegler (2006a, 2007a, b);  Kleeberg et al. (2004);  Lenartz et al. (2000);  Longhi et al. (2014, 2020);  Mabed et al. (2004);  Oei et al. (2018);  Pelzer et al. (2018);  Reynel et al. (2019, 2020);  Schad et al. (2018b);  Schumacher et al. (2003);  Seifert et al. (2007);  Steuer-Vogt et al. (2001);  Tröger et al. (2012, 2016);  Werthmann et al. (2017b, 2018a, b, d, 2019a);  Zaenker et al. (2012)  🡪 31 publications, 34 studies |
| 6 | Immunological parameters including Neuropenia and Body temperature | Elsasser-Beile et al. (2005a);  Enesel et al. (2005);  Gardin (2009);  Gorter et al. (1998);  Huber et al. (2002, 2011);  Kim et al. (2012);  Klose et al. (2003);  Loewe-Mesch et al. (2008);  Longhi et al. (2020);  Pelzer et al. (2018);  Schink et al. (2007);  Semiglasov et al. (2004), Semiglazov et al. (2006);  Son et al. (2010);  Steuer-Vogt et al. (2001);  Tröger et al. (2009, 2014b),  🡪 18 publications, 17 studies |
| 7 | Cost outcomes and Cost-effectiveness | Thronicke et al. (2020a)  🡪 1 publication, 1 study |
| 8 | Effect on side effects of conventional cancer therapy including body weight and cancer related fatigue and on disease related symptoms | Bar-Sela et al. (2013);  Bock et al. (2004a, 2014);  Beuth et al. (2008);  Cazacu et al. (2003);  Friedel et al. (2009);  Kim et al. (2012);  Klose et al. (2003);  Longhi et al. (2014)  Matthes et al. (2010);  Piao et al. (2004);  Schumacher et al. (2003);  Semiglasov et al. (2004), Semiglazov et al. (2006);  Steele et al. (2014a);  Tröger et al. (2013);  Wode et al. (2009);  Zaenker et al. (2012)  🡪 18 publications, 17 studies |
| 9 | Molecular activity of tumor reduction | Cazacu et al. (2003) (in vitro part of the study; in this article this part is not examined)  🡪 1 publication, 1 study |
| 10 | Impact on pleural effusion and aszites | Bar-Sela et al. (2006);  Cho et al. (2016), Cho and Kim (2018);  El-Kolaly et al. (2016);  Eom et al. (2017, 2018);  Gaafar et al. (2014);  Lee et al. (2019);  Oh (2020)  🡪 9 publications, 9 studies |

The numbers of the references refer to the reference list in the main manuscript.
